# Luciferase Expression Allows Bioluminescence Imaging But Imposes Limitations on the Orthotopic Mouse (4T1) Model of Breast Cancer

**DOI:** 10.1038/s41598-017-07851-z

**Published:** 2017-08-10

**Authors:** V. P. Baklaushev, A. Kilpeläinen, S. Petkov, M. A. Abakumov, N. F. Grinenko, G. M. Yusubalieva, A. A. Latanova, I. L. Gubskiy, F. G. Zabozlaev, E. S. Starodubova, T. O. Abakumova, M. G. Isaguliants, V. P. Chekhonin

**Affiliations:** 1 Research and Education Center for Medical Nanobiotechnology, Pirogov Russian National Research Medical University, Ministry of Health of the Russian Federation, Moscow, Russia; 2 Federal Research and Clinical Center of Specialized Medical Care and Medical Technologies, Federal Biomedical Agency of the Russian Federation, Moscow, Russia; 3 Department of Microbiology, Tumor and Cell Biology, Karolinska Institutet, Stockholm, Sweden; 4 Department of Fundamental and Applied Neurobiology, Serbsky National Research Center for Social and Forensic Psychiatry, Ministry of Health of the Russian Federation, Moscow, Russia; 5 Engelhardt Institute of Molecular Biology, Russian Academy of Sciences, Moscow, Russia; 6 Chumakov Federal Scientific Center for Research and Development of Immunobiological Preparations, Moscow, Russia; 7 N.F. Gamaleya Research Center of Epidemiology and Microbiology, Moscow, Russia; 8 Riga Stradins University, Riga, Latvia

## Abstract

Implantation of reporter-labeled tumor cells in an immunocompetent host involves a risk of their immune elimination. We have studied this effect in a mouse model of breast cancer after the orthotopic implantation of mammary gland adenocarcinoma 4T1 cells genetically labelled with luciferase (Luc). Mice were implanted with 4T1 cells and two derivative Luc-expressing clones 4T1luc2 and 4T1luc2D6 exhibiting equal *in vitro* growth rates. *In vivo*, the daughter 4T1luc2 clone exhibited nearly the same, and 4T1luc2D6, a lower growth rate than the parental cells. The metastatic potential of 4T1 variants was assessed by magnetic resonance, bioluminescent imaging, micro-computed tomography, and densitometry which detected 100-μm metastases in multiple organs and bones at the early stage of their development. After 3–4 weeks, 4T1 generated 11.4 ± 2.1, 4T1luc2D6, 4.5 ± 0.6; and 4T1luc2, <1 metastases per mouse, locations restricted to lungs and regional lymph nodes. Mice bearing Luc-expressing tumors developed IFN-γ response to the dominant CTL epitope of Luc. Induced by intradermal DNA-immunization, such response protected mice from the establishment of 4T1luc2-tumors. Our data show that natural or induced cellular response against the reporter restricts growth and metastatic activity of the reporter-labelled tumor cells. Such cells represent a powerful instrument for improving immunization technique for cancer vaccine applications.

## Introduction

Breast cancer (BC) is the leading cause of female oncological mortality and the second leading cause of death in the general population. The five-year survival rate of BC patients is 66%; remote metastases are found in some patients five or more years after removal of the primary focus^1^. BC metastasizes most frequently to the lungs, liver, and bones^2–4^, reducing life expectancy to two years^5, 6^. Severe clinical manifestations and the lack of effective methods for treating and preventing the metastasis formation make the development and testing of new therapeutic technologies against breast cancer an urgent task.

Preclinical trials of these technologies require adequate experimental models. The best for BC is the orthotopic implantation of tumor cells into syngeneic immunocompetent mice. It reproduces the entire pathogenic cycle, including formation of the primary orthotopic focus, involvement of regional lymph nodes, and development of remote metastases^7, 8^, including metastasis in bones^4, 9^. A powerful representative of these models is a rapidly growing and highly metastatic murine adenocarcinoma 4T1 derived from a spontaneous mammary tumor in a BALB/c mouse^4, 10^. Injected into the fat pad of a mammary gland, 4T1 cells metastasize to lungs, liver, bone, and brain. Subclones of 4T1 cells exhibiting various degrees of metastatic dissemination are employed to define gene expression patterns typical to primary tumors, lymph node colonization and metastatic outgrowth in lymph nodes, as well as distant organ metastases^8^. Subclones of 4T1 cells stably transfected with reporter genes have been developed and applied for optical monitoring of primary tumors, metastases and circulating tumor cells^9, 11–13^. Implantation of the reporter-labeled tumor cells in an immunocompetent host involves a risk of their immune elimination due to the xenogeneic nature of the reporters^14, 15^. Anti-reporter immune response can alter both the invasiveness and the metastatic potential of the labelled tumor cells. Here, using a combination of MRI, bioluminescent *in vivo* imaging, and micro-computed tomography (micro-CT), we evaluated the ability of orthotopic 4T1 cell lines expressing luciferase to form primary tumors and develop metastases in various organs and tissues. We found that luciferase expression affects the propensity of 4T1 cells to form metastases, specifically in the brain, related this phenomenon to the immunogenicity of the reporter presented by the tumor cells, and demonstrated the usefulness of this phenomenon in development of cancer vaccine technologies.

## Results

### Comparative characteristics of the Luc-expressing 4T1 clones *in vitro*: growth rate and level of reporter expression

**Figure 1:**
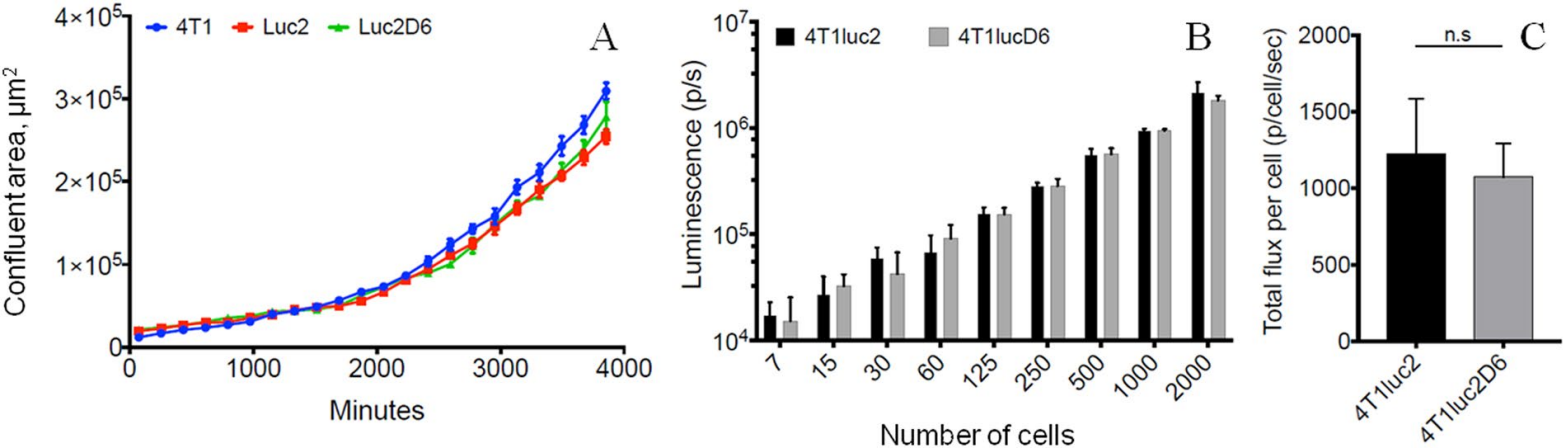
Growth rates of the murine adenocarcinoma 4T1 cell line and its sublclones expressing luciferase 4T1luc2D6 and 4T1luc2. The time course of the proliferation of the parental 4T1, and 4T1luc2D6 and 4T1luc2 (PerkinElmer) clones as determined using Nikon Biostation CT (Nikon, Japan) with STDV (**A**); Bioluminescence from 4T1luc2 and 4T1luc2D6 cells in culture assessed by bioluminescent imaging (Spectrum CT, Perkin Elmer) (**B**); Average level of bioluminescence of 4T1luc2 and 4T1luc2D6 cells (photons/cell/sec) (**C**). Results from five independent measurements. No significant difference between any of the analysed parameters (p > 0,05; Mann Whitney test).

The starting point was to characterize the growth rate and level of reporter expression of luciferase (Luc) expressing 4T1luc2D6^16^ and commercially available 4T1luc2 clone (ref. 17 Perkin Elmer) as compared to the parental 4T1 cells. All three demonstrated similar growth rates *in vitro* (Fig. 1A) corroborating the earlier findings^18^. Luc activity in 4T1lucD6 and 4T1luc2 cells was measured as the bioluminescence of a fixed number of cells taken in the range from 5 to 2000 (Fig. 1B). Luc activity per cell was calculated as an average number of photons released per 4T1luc2D6 or 4T1luc2 cell. In the early passage, 4T1lucD6 cells and 4T1luc2 cells released 1071 ± 220 and 1225 ± 357 photons/sec/cell, respectively, i.e. had similar levels of Luc activity (Fig. 1B,C). According to Conti *et al*.^19^, one Luc molecule releases one photon per sec, thus, an average 4T1lucD6 and 4T1luc2 cell expressed from 1000 to 1200 reporter molecules.

### Comparative *in vivo* characteristics of the Luc-expressing 4T1 clones: induction of the primary tumor focus

**Figure 2:**
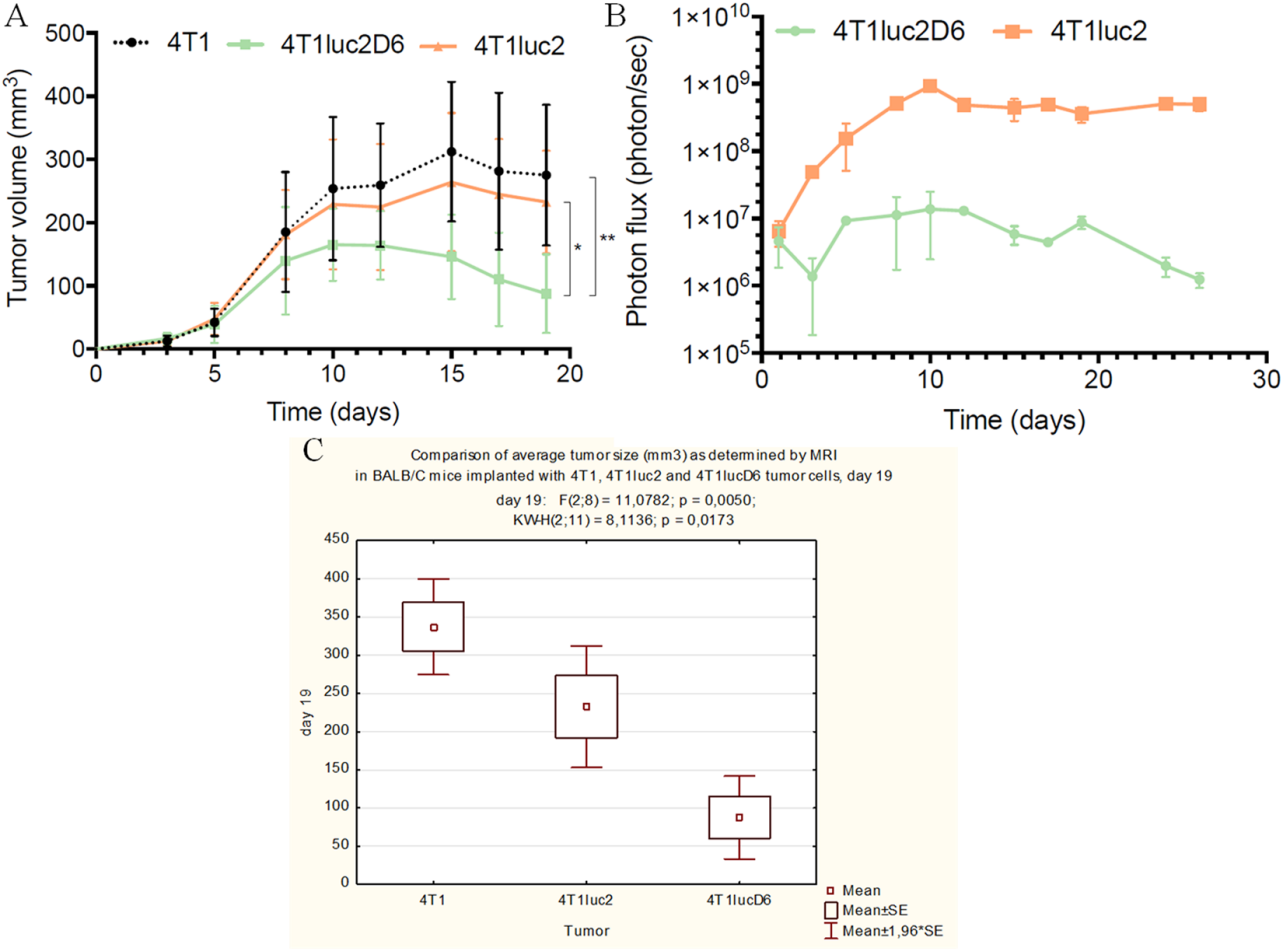
Growth in BALB/c mice (n = 5–6 per group) of primary tumors induced by the implantation of 4T1, 4T1luc2 and 4T1lucD6 cells (see Methods for description). Growth curves obtained by MRI visualize average tumor volume in cubic mm, with STDV (**A**); Growth of primary tumors induced by implantation of 4T1luc2 and 4T1lucD6 cells monitored by bioluminescent imaging; curves depict an average photon flux from the tumor per sec, with STDV (**B**); Statistical comparison of the tumor sizes evaluated by MRI (**C**). Median size of 4T1lucD6 tumors is significantly lower (p = 0,017), and of 4T1luc2 tumors tend to be lower than of 4T1 tumors, although the difference is not significant (p = 0,17). Day of implantation is counted as day 0. Statistical comparisons are done using Kruskal Wallis and Mann-Whitney tests (Statistica AXA 10.0).

Upon implantation, 4T1, 4T1luc2D6 and 4T1luc2 cells formed solid tumors, palpated by day 7 as a firm mass approximately 0.5 cm in diameter. By the experimental end-point, tumor areas reached a size of 1000 mm^2^, some expanding into the anterior lateral wall of the chest. The morphometric analysis demonstrated that tumor volume in mice inoculated with Luc-expressing subclone 4T1luc2D6 increased 1.5–2.0 times slower than in mice inoculated with the parental 4T1 cells (Fig. 2A). Tumors formed by 4T1luc2 cells grew similarly to the tumors formed by the parental cells, although by week 2 their size tended to be lower than the size of the parental 4T1 tumors (Fig. 2C). Both MRI and bioluminescent imaging of the sites of implantation of tumor cells confirmed poor growth characteristics of 4T1luc2D6 compared to both 4T1 and 4T1luc2 cells (Fig. 2A–C). Thus, although all three 4T1 clones had identical *in vitro* proliferation rates and a similar propensity to form primary tumors, the growth rate of the Luc-expressing tumors, specifically of the ones formed by 4T1luc2D6 clone, was lower than that of the tumors formed by the parental 4T1 clone. Interestingly, bioluminescent imaging (BLI) measurements indicated up to 100-fold, whereas MRI, only two-fold difference in the size of 4T1luc2 and 4T1luc2D6 tumors (Fig. 2A,B).

### Evaluation of Luc activity in the resected tumors

**Figure 3:**
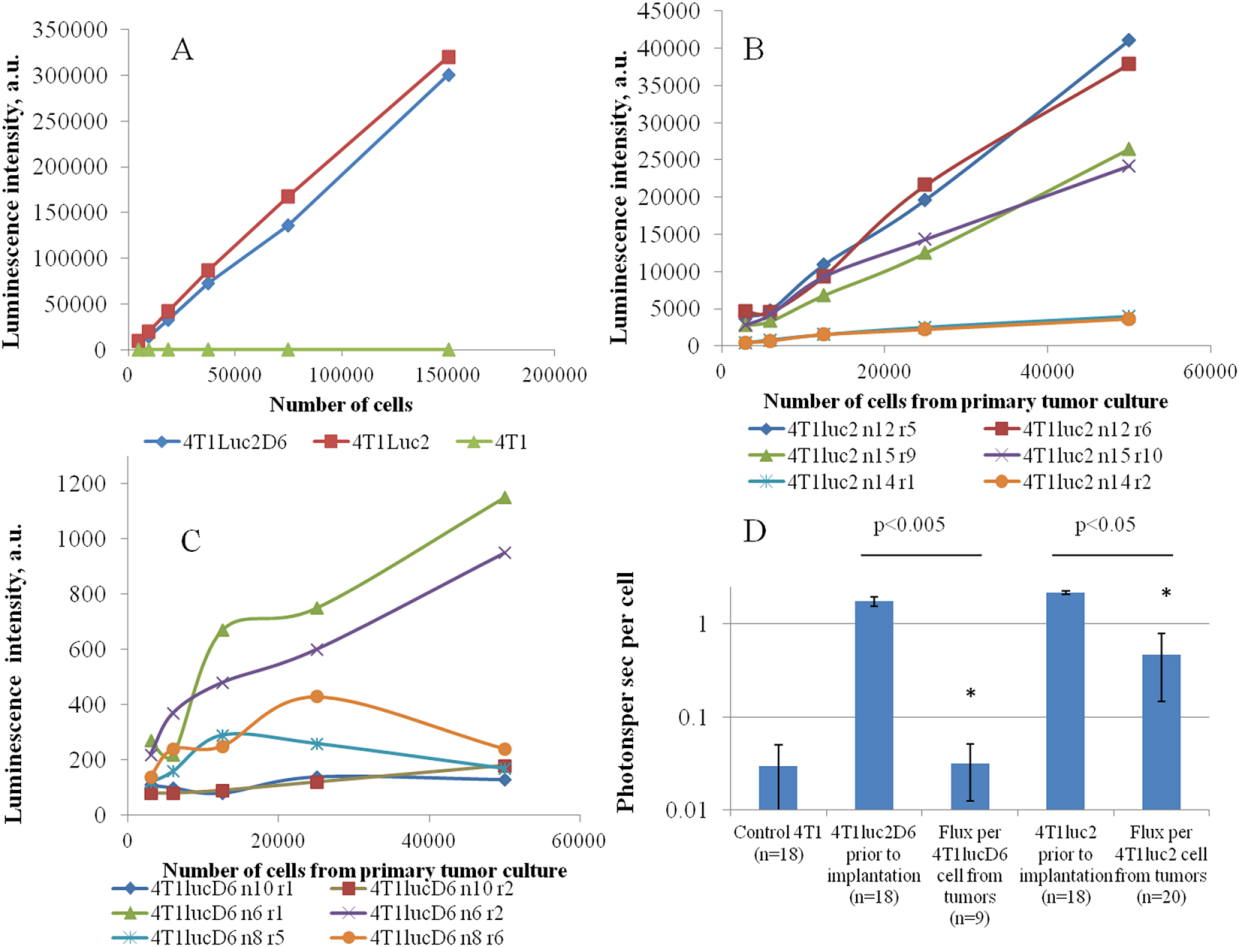
Comparison of the luciferase activity measured as the intensity of luminescence (in arbitrary units a.u.; Enspire, Perkin Elmer) in the original 4T1luc2 and 4T1luc2D6 cell lines and in cell cultures prepared from the 4T1luc2 and 4T1luc2D6 tumors by the experimental end-point. Luciferase activity in the 4T1luc2 and 4T1luc2D6 cells prior to implantation (**A**); Luciferase activity in primary cell cultures prepared from tumors formed in BALB/c mice by implantation of 4T1luc2D6 (n = 3; 6 tumors) (**B**) or 4T1luc2 cells (n = 3; 6 tumors) (**C**); Recalculation of the average luciferase activity per cell with STDV (**D**). Individual tumors are coded by the cell line, mouse and tumor numbers, for example “4T1luc2D6 n10 r1” designate a tumor caused by implantation of cell line 4T1luc2D6 into mouse No. 10 and refer to sample No. 1 from one of the tumors. Curves in panels B and D show dependence of luciferase activity on the number of cells used in the assay; data represent the average of three parallel measurements. Statistical comparisons are done using Mann-Whitney test (Statistica AXA 10.0); **p < 0,01, and *p < 0,05.

Intrigued by the discrepancy of MRI and BLI assays with respect to the size of Luc-expressing tumors, we launched the evaluation of Luc activity per cell by the experimental end-point reached within three weeks post implantation. For this, tumors (5–6 per tumor type) were excised, sheered, digested by collagenase, and run through cell strainers to establish single cell cultures. Primary cell cultures were then assessed for luminescence intensity (ExPire, Perkin Elmer). Interestingly, 4T1luc2D6 cells were found to have 60-fold lower luminescence intensity compared to the cell lines used for implantation (Fig. 3; p < 0,0001; Difference test). Luminescence intensity in the 4T1luc2 tumors had also decreased, but only four-fold (Fig. 3; p < 0,0001). Since the luminescence intensity directly correlates to the photon flux per cell (Suppl. Fig. S1) the data from the primary cultures of tumor-derived 4T1luc2D6 and 4T1luc2 cells indicated a 60- and, respectively, 4-fold decrease of the luciferase expression in these cells compared to the implanted cell lines.

Moreover, the results of assessment of Luc activity in the primary cultures of tumor-derived 4T1luc2D6 cells demonstrated the heterogeneity of the tumors. Primary tumor cells from some of these mice emitted very weak luminescence (as 4T1luc2D6 cells explanted from mouse #10; 4T1luc2D6-#10; Fig. 3C). Furthermore, little increase in bioluminescence was observed with an increase in the amount of cells in the luminescence test with concordant data demonstrated by cells originating from two tumors in one and the same mouse (for example, 4T1luc2D6-#8; Fig. 3C). Tumors formed by 4T1luc2 cells were more homogenous, as could be seen from a linear dependence of the luciferase activity on the number of primary cells taken into the assay (see data for mice #12 and 15; Fig. 3D). Still some of the mice/tumors had a reduced luciferase activity with concordant data for tumors originating from one and the same mouse (for example, 4T1luc2-#14; Fig. 3D). Assays of luciferase activity demonstrated that, contrary to what is normally observed in 4T1luc2 cells *in vitro* ^17^ after three weeks of *in vivo* growth, all Luc-expressing cells reduced the levels of reporter expression. The reduction was more drastic for cell line 4T1luc2D6 obtained by stable transfection, and less pronounced for cell line 4T1luc2 obtained by lentiviral transduction, but it was significant in both cases, accounting for inaccurate estimation of tumor growth if the latter is performed by BLI only.

### The metastatic potential of 4T1 cells expressing luciferase compared to the parental cells

#### Multi-modal detection of metastasis of 4T1 and 4T1luc tumors

**Figure 4:**
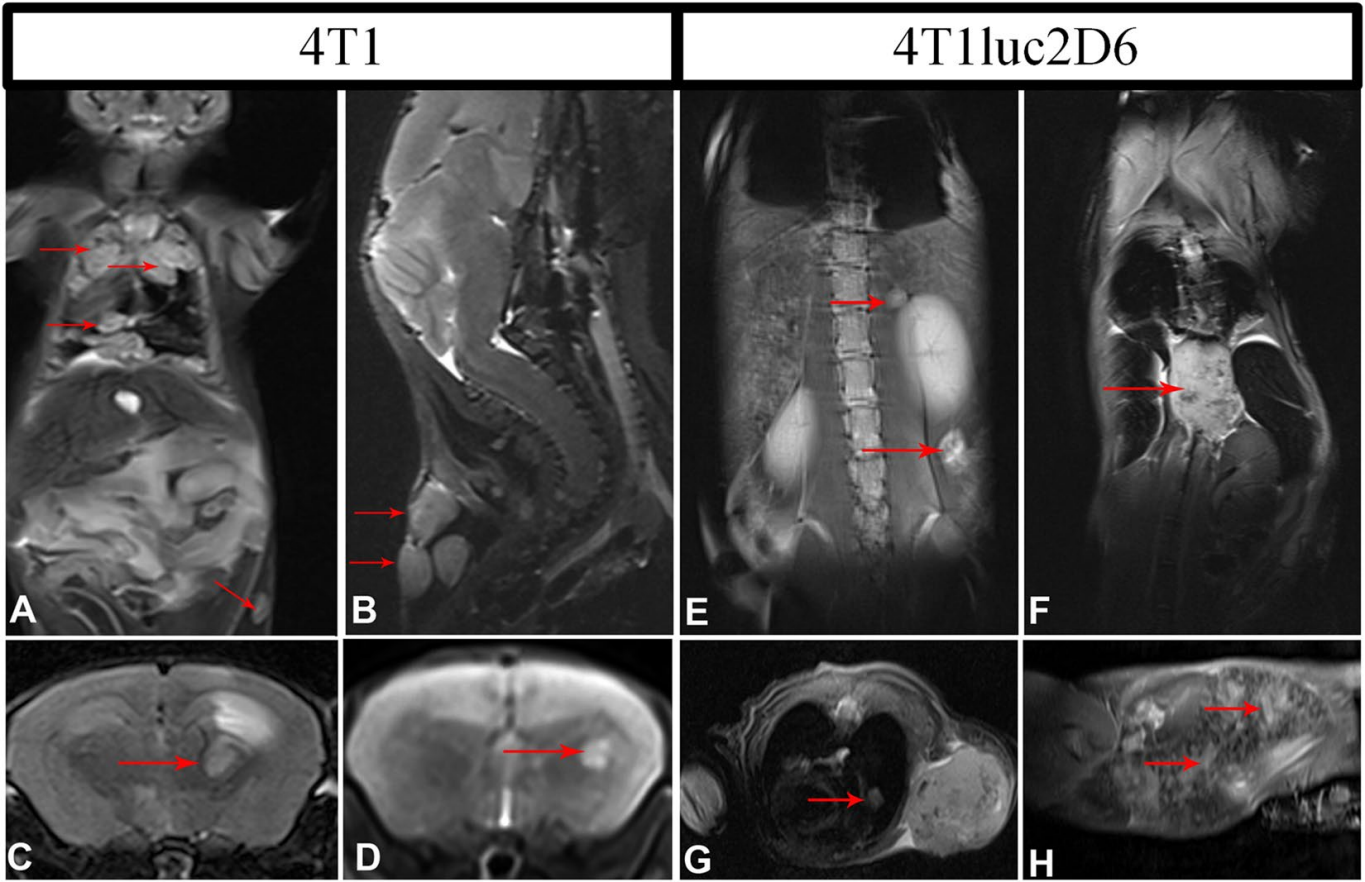
Multiple metastases in BALB/c mice after the orthotopic implantation of 4T1 (**A**–**D**) or 4T1luc2D6 cells (**E**–**H**) as visualized by the T2-weighted MRI. 4T1 metastasis in lungs, the lower arrow indicates the metastasis in a lymph node, transversal projection (**A**); Multiple metastatic lesions of the 4T1 primary tumor into the posterior cervical lymph nodes (indicated by arrows), sagittal projection (**B**); Metastatic lesions induced by 4T1 cells in the brain (indicated by arrow) in coronary projections, metastases are manifested by massive perifocal edema (**C**,**D**); Metastases of 4T1luc2D6 tumor in the retroperitoneal and paravertebral regions, transversal projections (**E** and **F**, respectively); Single metastasis of the 4T1luc2D6 tumor in the lung, coronary projection (**G**); Metastases of the 4T1luc2D6 tumor in the spleen (indicated by arrows), sagittal projection (**H**).

The metastatic lesions in the 4T1-implanted mice were detected by high-resolution MRI (Fig. 4). MRI revealed multiple metastases of 4T1 tumors in the organs of the thoracic and abdominal cavities, brain, and soft tissues of the neck and legs (Fig. 4A–D). For Luc-expressing tumors, MRI results were supported by 2D/3D BLI (Spectum CT), which reliably detected the thoracic metastases of 4T1luc2D6 sized 500 μm, and the metastases occurring in surface soft tissues of the head and legs as small as 200 μm (Fig. 4). Severe metastatic lesions were detected in the thoracic organs. Paravertebral spread of the tumor was also observed (resulting in some cases in tumor growth into the vertebrae). No remote metastases were found in mice implanted with 4T1luc2 cells (data not shown).

**Figure 5:**
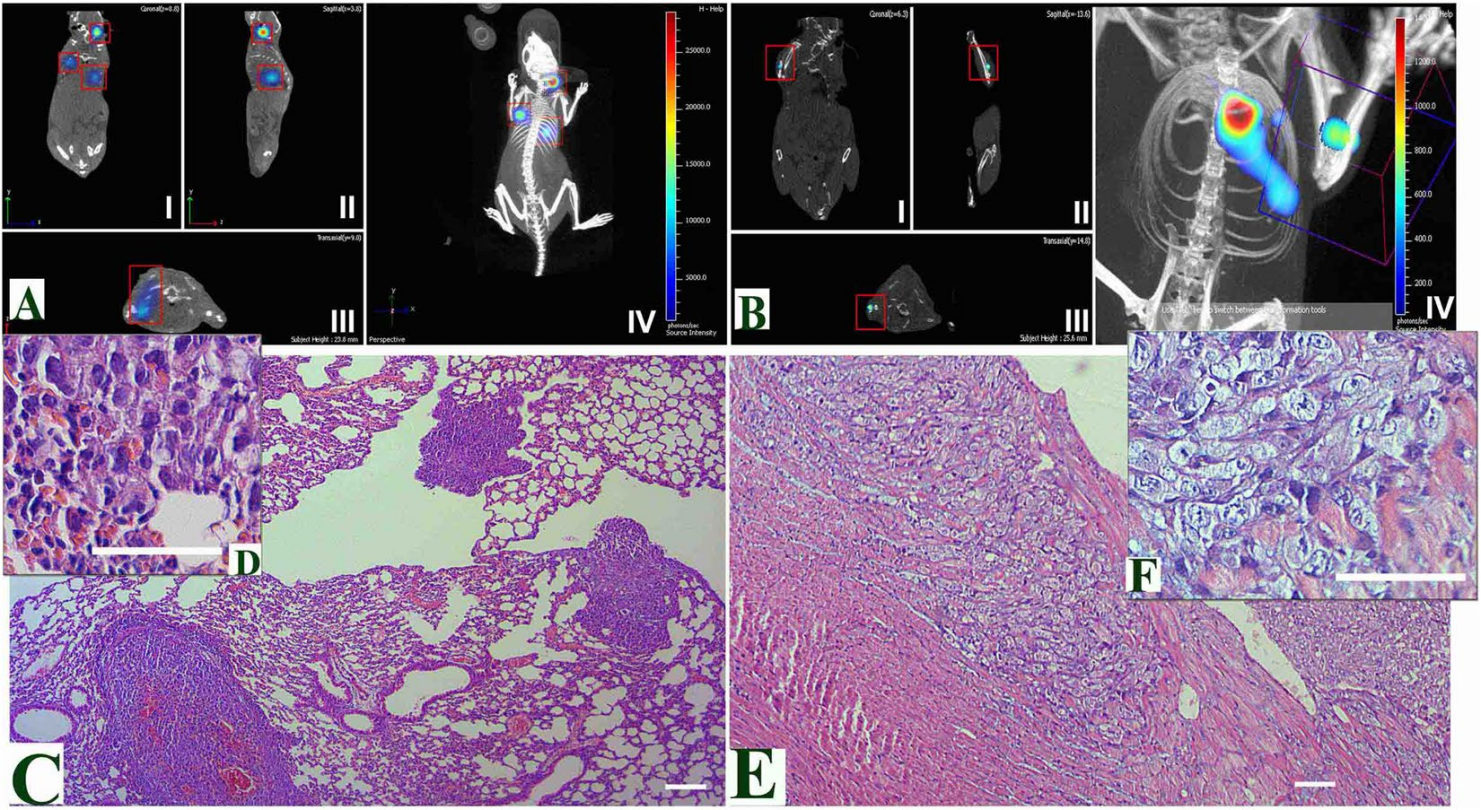
Characteristics of the metastatic potential of Luc-expressing 4T1 cells in the orthotopic breast cancer model evaluated by BLI and confirmed by MRI. Small metastases of 4T1luc2D6 cells in the lungs and soft tissues one week after removal of the primary focus (week 4 after the implantation of 4T1lucD6 cells) visualized by micro-CT, 3D BLI (**A**,**B**), and verified by histochemistry (**C**,**D**,**E**,**F**). Metastasis as a single source of luminescence are detectable in the lung and neck (**A**), and in the lung and muscle tissues of the lower right foreleg (**B**), panels I, II, III demonstrate microCT xy-, yz-, and zx- projections respectively, and panel IV, 3D reconstruction of microCT and BLI (**A**,**B**); Histological verification of the multiple 200–500 μm metastasis in lungs (**C**,**D**), and muscle tissues (**E**,**F**); Metastases in muscle tissue, 300 × 600 μm in size, detected by bioluminescence imaging (**E**). Hematoxylin-eosin staining of sections of paraffin-block preparations of the lung metastases (**C**,**D**) and metastases in muscle tissue (**E**,**F**) demonstrated hypercellular foci consisting of the polymorphic cells characteristic of the high-grade carcinomas. Magnification 50× (**C**,**E**) and 200× (**D**,**F**); scale bar 50 μm.

Neither 2D BLI, nor 3D reconstruction could detect small metastases formed by Luc-expressing 4T1 cells near the intensely luminescent primary tumor. To detect small metastases we used the surgery-driven enhancement of metastasis development^20^. This methodology is very sensitive for detecting even the smallest dormant metastases missed by the standard visualization techniques. One week after removal of the primary focus, mice implanted with 4T1luc2D6 cells exhibited multiple metastases in the lungs and the soft tissues of the head, legs, and thoracic cavity (Fig. 5A–F). Interestingly, combination of assessments done before and after the resection of primary tumors demonstrated that mice with 4T1lucD6 tumors had significantly fewer metastases in the internal organs and soft tissues than mice implanted with the parental 4T1 cells (Table 1). Specifically, they had fewer metastases in lungs, a tendency to fewer metastases in liver, and no detectable metastases in the brain (Table 1).

**Table 1:** Characteristics of the metastatic potential of 4T1 and 4T1luc2D6 cells in the orthotopic breast cancer model evaluated by BLI and confirmed by MRI.

Mice (n = 20) implanted with	Total metastases	Lymph nodes	Lungs	Spleen	Liver	Bones	Brain	Average n, number of metastases per mouse
4T1	54	19	15	7	6	4	3	11.4 ± 2.1
4T1luc2D6	31	17	7^*^	3	2^(*)^	2	0	4.5 ± 0.6^**^

**p < 0,00001 (t-test); ^*^p < 0.05 and ^(*)^p < 0.1 (U-test).

### Detection of metastases in skeletal bones

**Figure 6:**
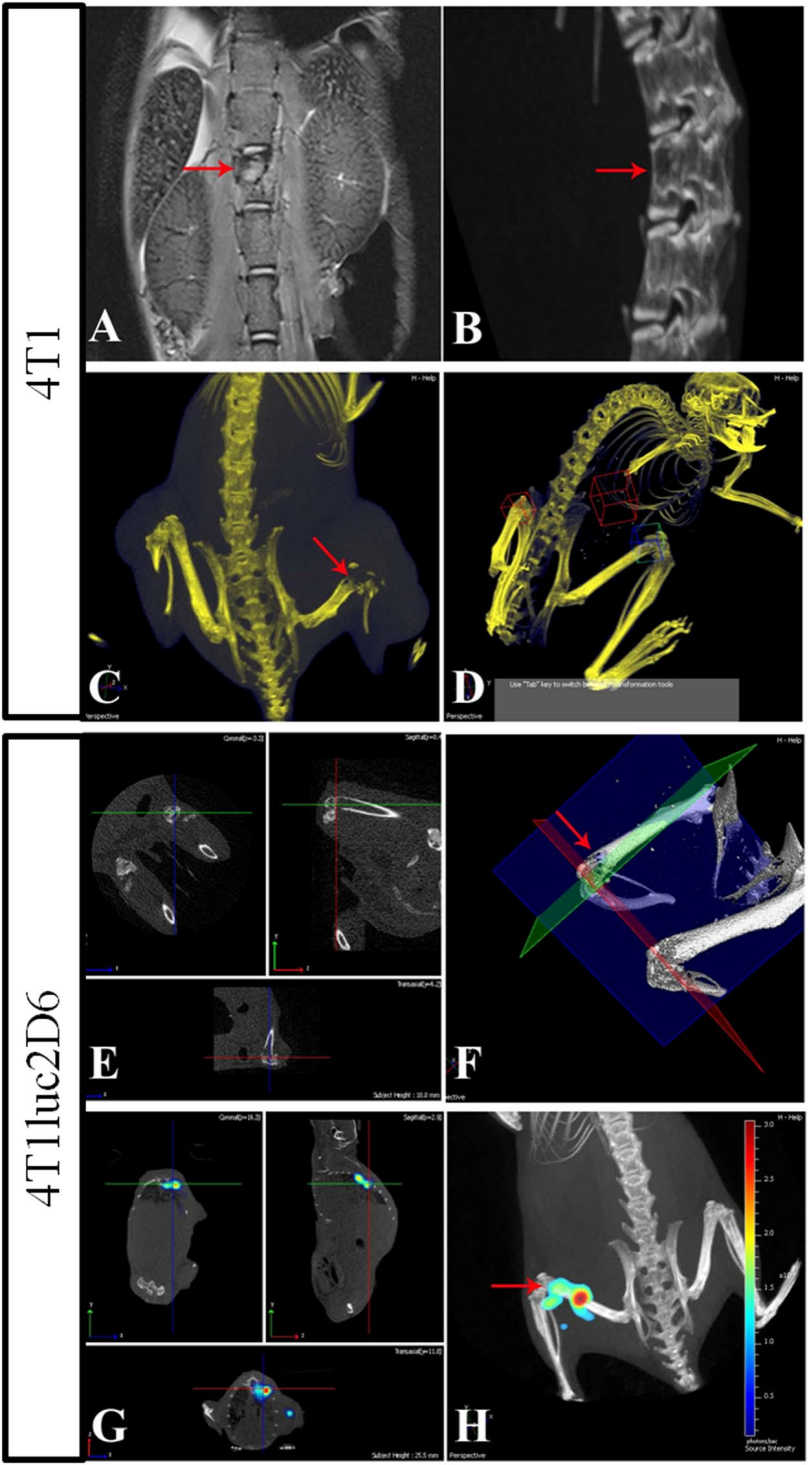
Bone metastases formed by 4T1 (**A**–**D**) and 4T1luc2D6 (**E**–**H**) tumors at week 4 after implantation visualized by the combination of MRI, micro-CT and optical imaging. The T2-weighted MRI of a mouse with a 4T1 carcinoma, coronary section, the arrow indicates a metastasis in the vertebral body (**A**); Targeted high-resolution micro-CT of the same mouse with a fragment of 3D reconstruction, the arrow indicates the focus of osteolysis of the vertebral body (**B**); Micro-CT of a mouse with a 4T1 carcinoma on day 28 after implantation, the arrow indicates a osteolytic lesion by metastasis of the distal epiphysis of the right femur (**C**); Micro-CT of a 4T1 mouse with a metastasis in the region of the right knee-joint, the areas subjected to densitometry are highlighted (**D**); Targeted micro-CT of a 4T1luc2D6 mouse, the arrow indicates the metastasis in the distal epiphysis of the right femur (**E**,**F**); 4T1luc2D6 metastasis in the vertebral body (**G**) and the lower third of the left femur (**H**) visualized by bioluminescent imaging.

The parental 4T1 clone has a marked capacity to form metastases in bones, primarily detected by high-resolution MRI. In case of suspected metastases in the bone tissues, a targeted high-resolution micro-CT was performed followed by densitometry to confirm bone resorption. In this way, we detected the 0.5 × 0.8 mm metastasis in the body of the third lumbar vertebra (Fig. 6A,B) and a 0.4 × 0.6 mm metastasis in the distal epiphysis of the femur (Fig. 6C,D). Bone metastases in mice implanted with the Luc-expressing tumor cells were first revealed by 3D BLI (Fig. 6G,H). This technique reliably detected the 0.4 mm metastases to the vertebral bodies and epiphyses of large tubular bones (Fig. 6E–H).

We have developed a micro-CT protocol for Spectrum CT-driven densitometry of the large joints to detect early signs of bone resorption. For this, we located and normalized 3D objects in the reconstructed 3D image and determined the absolute average and relative densities within these objects in Hounsfield units (Fig. 6F). This technique detected a 0.3 × 0.5 mm metastasis in the knee joint of a 4T1-inoculated mouse (field of view, FOV of 6 × 6 cm). The lesion was not discernible at a standard micro-CT resolution. However, the averaged density in the affected epiphysis (normalized to the number of voxels in the selected 3D area) was at least two times lower than normal (1.41 × 10^2^ compared to 4.07 × 10^2^ Hounsfield units, respectively). Presence of the bone metastasis was confirmed by high-resolution micro-CT (FOV = 2.4 × 2.4 × 2 cm) (Fig. 6E–H). The latter demonstrated the applicability of the protocol for the detection of bone metastases at the earliest stages of bone destruction. Using this protocol, we demonstrated that the capacity of 4T1luc2D6 cells to metastasize into the skeletal bones was similar to that of the parental 4T1 cells. At the same time, we were unable to detect bone metastases in mice harboring 4T1luc2 tumors.

### Detection of metastases in brain

MRI detected brain metastases in 3 of the 20 mice in the 4T1 group. The metastases were accompanied by a severe mass effect and hemispheric edema (Fig. 4C,D), and diffuse neurological symptoms. At the same time, BLI revealed no brain metastases in mice implanted with either 4T1luc2D6, or 4T1luc2 cells. One brain lesion was detected in a 4T1luc2D6-implanted mouse by MRI; however, it was not confirmed by the histological examination. The primary focus was removed from ten mice implanted with either 4T1luc2D6 (n = 5) or 4T1luc2 cells (n = 5). Four weeks later, mice were euthanized and all organs were removed for histological examination. Analysis of brain slices of two 4T1luc2D6 mice revealed perivasal metastases <100 μm in diameter (Supplementary Fig. S2) not detected by either BLI, or MRI. The fact that these metastases had not expanded after the removal of primary focus indicated that they were dormant and as such, were not recorded. No other brain metastases were revealed in either 4T1luc2- or 4T1luc2D6-implanted mice by any of the methods in use. Thus, the array of metastasis detection methods revealed that 4T1luc2D6 had a decreased and 4T1luc2 cells, low metastatic potential compared to the parental 4T1 cells (Table 1; no metastasis in 4T1luc2 implanted mice were detected in three weeks post the implantation). Specifically, Luc-expressing 4T1 cells were deficient in the capacity to metastasize in the brain.

### Immunogenicity of luciferase in the context of the luciferase expressing tumor

**Figure 7:**
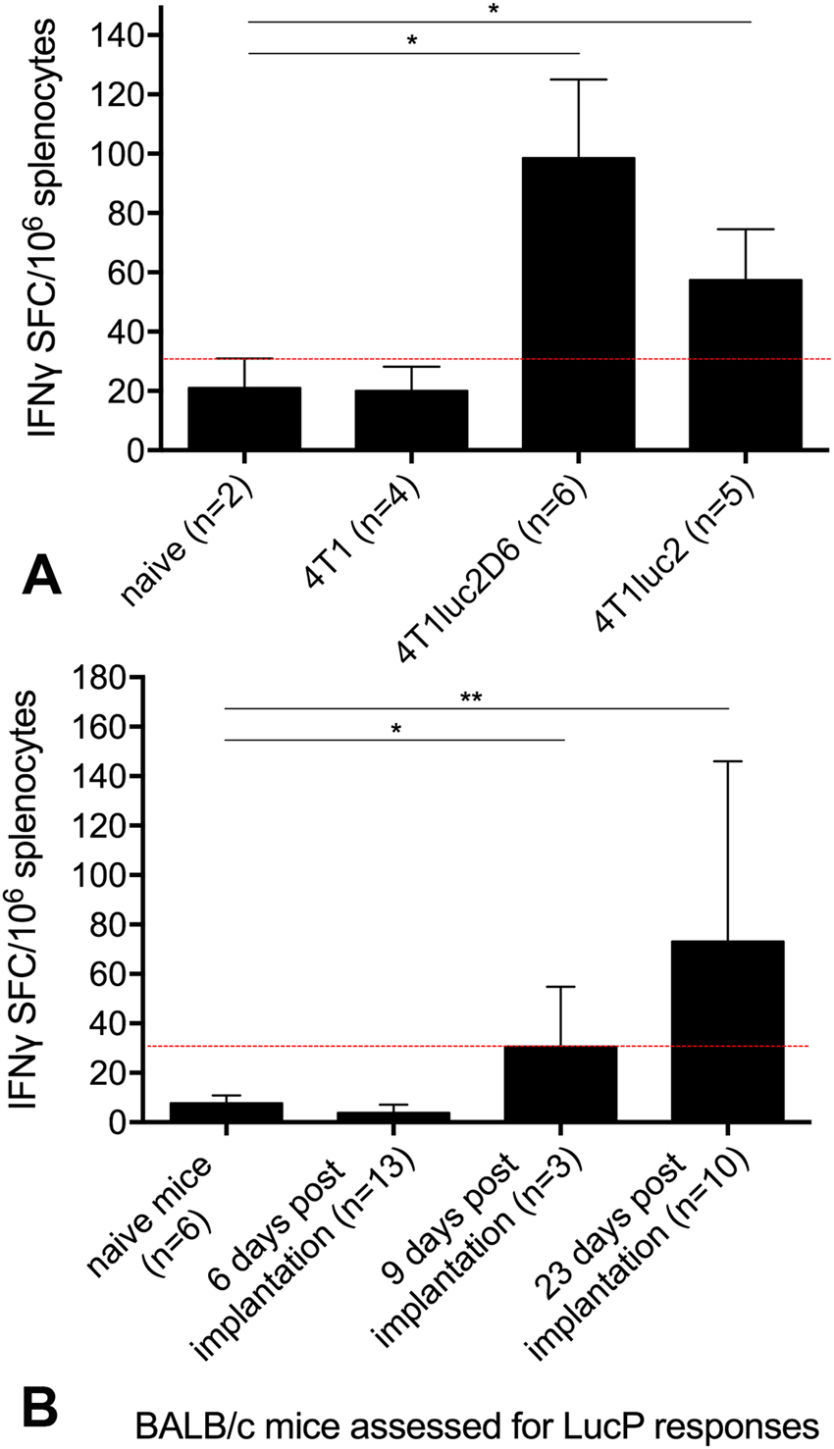
*In vitro* IFN-gamma response of lymphocytes of BALB/c mice implanted with Luc-expressing 4T1luc2D6 or 4Tl1luc2, or parental 4T1 cells, to stimulation with peptide GFQSMYTFV representing an immunodominant CTL epitope of luciferase (LucP) assessed using IFN-γ ELISpot (Mabtech). IFN-γ response to LucP by the experimental end-point assessed as the average number of IFN-γ spot forming cells per mln splenocytes (SFC/mln) with STDV; *p < 0.05, IFN-γ response to LucP in BALB/c mice implanted with 4T1luc2D6 and 4T1luc2 cells compared to IFN-γ response exhibited by naïve or 4T1-implanted mice (Mann-Whitney test) (**A**); Development of cellular immune response to LucP in mice implanted with 5000 4T1luc2 cells, on days 6, 9 and 23 post implantation; *p < 0.05, IFN-γ response to LucP in BALB/c mice implanted with 4T1luc2 cells on days 9 and 23 post implantation compared to IFN-γ response exhibited by naïve mice or mice implanted by 4T1luc2 and assessed on day 6 post implantation (Friedman ANOVA and Kendall Coeff. of Concordance) (**B**). Red line indicates the cut-off for the specific IFN-γ response, defined as an average number of lymphocytes producing IFN-γ in response to stimulation with LucP ± 3 STDV per mln lymphocytes. Cut-off was established in independent *in vitro* tests done on splenocytes of naïve mice (n = 5).

We hypothesized that the aberrant capacity of Luc-expressing tumor cells to form metastases may be a result of an immune response against luciferase. We made a head-to-head comparison of the immunogenicity of these cells lines by collecting spleens from all mice, also implanted with the parental 4T1 cells, isolating splenocytes, and stimulating them with a synthetic peptide representing the immunodominant CTL epitope of luciferase recognized by BALB/c mice (LucP)^21^. The number of reactive T-cells was assessed by IFN-γ ELISPOT (Mabtech; Fig. 7A,B). Luc-specific IFN-γ response was detected in both 4T1luc2D6- and in 4T1luc2-, but not in naïve, or 4T1-implanted mice (Fig. 7A). We followed the development of this response in mice implanted with 4T1luc2 cells in time. Lymphocytes were collected prior to and post 4T1luc2 implantation on days 6, 9 (PBMCs) and 23 (splenocytes), and stimulated with LucP. A weak LucP-specific production of IFN-γ first detected on day 9 post implantation, increased to significant levels by day 23 (Fig. 7B). Interestingly, induction of the response coincided with a decrease in the growth rate of the primary focus formed by 4T1luc2 cells (Fig. 2A).

### Immune response against luciferase induced by DNA immunization protects against the establishment of 4T1luc2 tumors

To further test whether a Luc-specific immune response can limit tumor invasiveness, we primed and then boosted mice with a plasmid (pVaxLuc, dubbed Luc DNA) encoding the same variant of firefly luciferase as is expressed by 4T1luc2 and 4T1lucD6 cells. Control mice received an empty vector (pVax1). Plasmids were delivered by intradermal injections. Immediately after, mice were electroporated over the injection sites using DermaVax (Cellectis) generating driving pulses of 100 V (as described)^22^ (n = 4) or CUY21 EditII (BEX Ltd.) generating driving pulses of 100 V (n = 6), or 50 V (n = 4).

**Figure 8:**
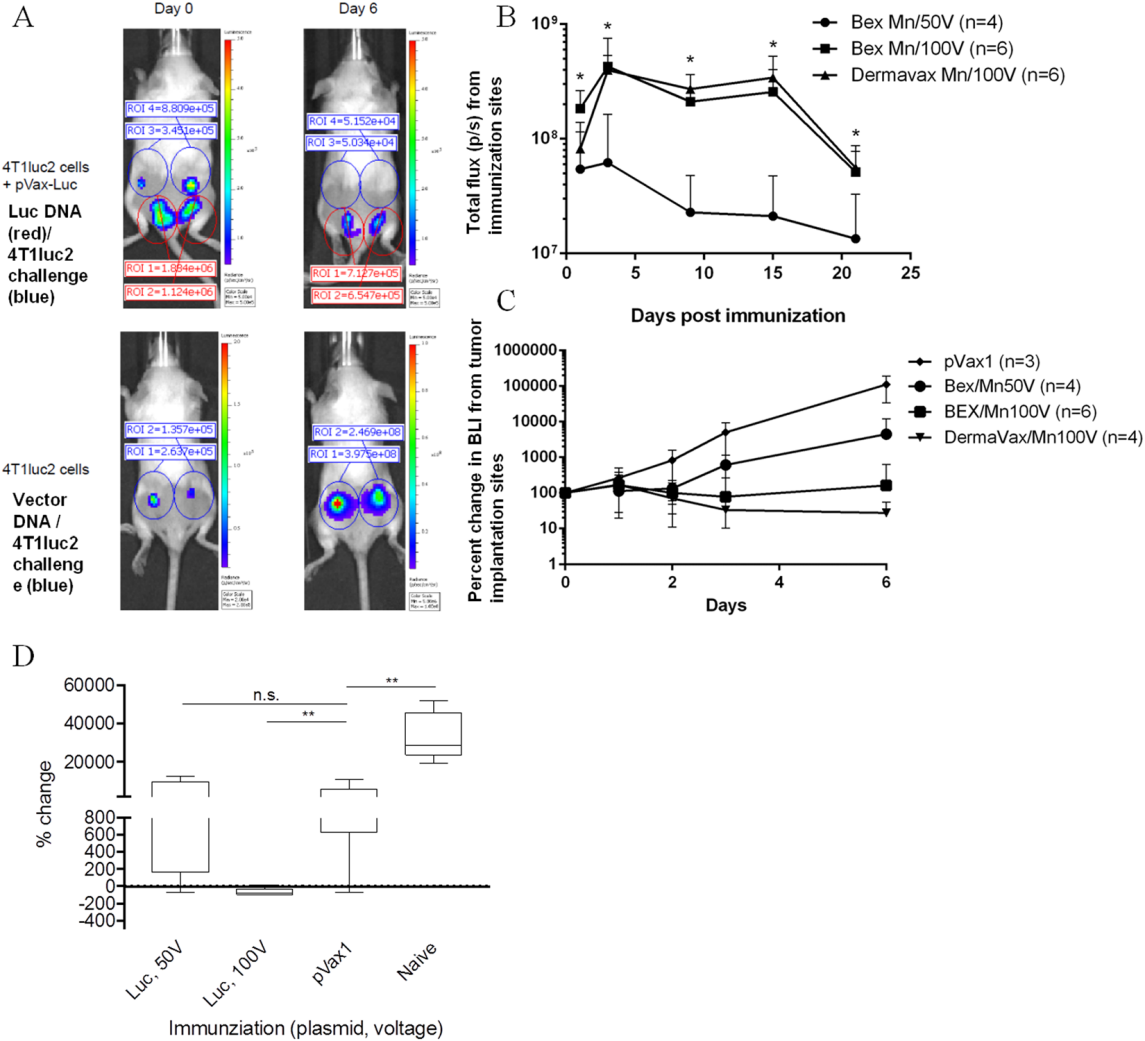
Intradermal Luc DNA immunization followed by electroporation (EP) with driving pulses of 100 V makes BALB/c mice resistant to initiation of 4T1luc2 tumors. BALB/c mice were immunized by plasmid encoding firefly luciferase pVaxLuc delivered by intradermal injections followed by EP using multineedle (Mn) electrodes mounted on Dermavax (Cellectis) (n = 4) or CUY21EditII (BEX Ltd) electroporators at 100 V (n = 6) or 50 V (n = 4). Immunization (red circles) and 4T1luc2 implantation sites (blue circles) visualized by *in vivo* imaging (Spectrum CT) on days 1 and 6 post tumor cell implantation (43 and 48 days of the immunization cycle). Mice immunized with Luc DNA (A, upper panel) versus empty vector (A, lower panel). Text boxes demonstrate total flux from the respective regions of interest (ROI) (photons/sec) (**A**); Quantification of bioluminescence emission (BLI; photon flux/sec) from immunization sites illustrates the efficacy of Luc gene transfer and expression. From day 5, Luc DNA immunized mice electroporated at 100 V emitted stronger bioluminescence than mice electroporated at 50 V (p < 0,05; Mann Whitney test), no bioluminescence from immunization sites in control mice receiving pVax1 (**B**); Quantification of bioluminescence (photon flux/sec) from 4T1luc2 implantation sites in mice immunized with Luc DNA and electroporated with DermaVax/Mn at 100 V (n = 4), BEX/Mn at 100 V (n = 6), BEX/Mn at 50 V (n = 4), and vector immunized mice (n = 3) on days 0 to 6 post challenge (**C**); Difference (in %) in BLI from 4T1luc2 implantation sites after six days of tumor growth in mice immunized with Luc DNA with EP at 100 V (DermaVax/Mn + BEX/Mn groups, 10 animals, dubbed “Luc, 100 V”), or 50 V (BEX/Mn, 4 animals, dubbed “Luc, 50 V”), vector immunized (n = 3) and naive mice (n = 5, from an independent experiment) (**D**). *p < 0,05; **p < 0,1 (Statistica AXA 10.0).

Two weeks after the boost, mice were challenged with 4T1luc2 cells. Implantation was performed ectopically to allow simultaneous monitoring of the sites of immunization and of tumor implantation (Fig. 8A). We reasoned that the site of implantation may influence the viability of migrating tumor cells, but not the tumor’s invasiveness. Indeed, in an independent experiment, we found the growth rates of 4T1luc2 cells to be similar for orthotopical and for ectopical implantations (Supplementary Fig. S3A). To detect the early effects of Luc gene immunization on the tumor growth rate, we challenged mice with 5.0 × 10^3^ 4T1luc2 cells. The choice of this cell dose was based on the growth rate observations. When implanted, 5.0 × 10^3^ 4T1luc2 cells exhibited the highest growth rate in the period preceding the establishment of a visible solid tumor, and also demonstrated the best viability as determined by the stability of the bioluminescence from the primary focus (Supplementary Fig. S3B).

All mice immunized with Luc DNA and electroporated at 100 V resisted the initial tumor growth (Fig. 8A,C). By day 9, the protected animals retained only 15% of the initial bioluminescence signal from the implanted 4T1luc2 cells (Fig. 8C). The electroporation regimen with 50 V pulses promoted lower levels of luciferase expression than the regimen employing 100 V pulses (Fig. 8A,B), and conferred the protection to only one animal (Figs 8C and S4). A single mouse that resisted tumor growth (R) demonstrated a strong expression of Luc at the site of immunization both after the prime and after the boost (Supplementary Fig S4A,B); in nonresistant mice (NR) the expression tended to be weaker (p < 0.1; Supplementary Fig S4A–C).

**Figure 9:**
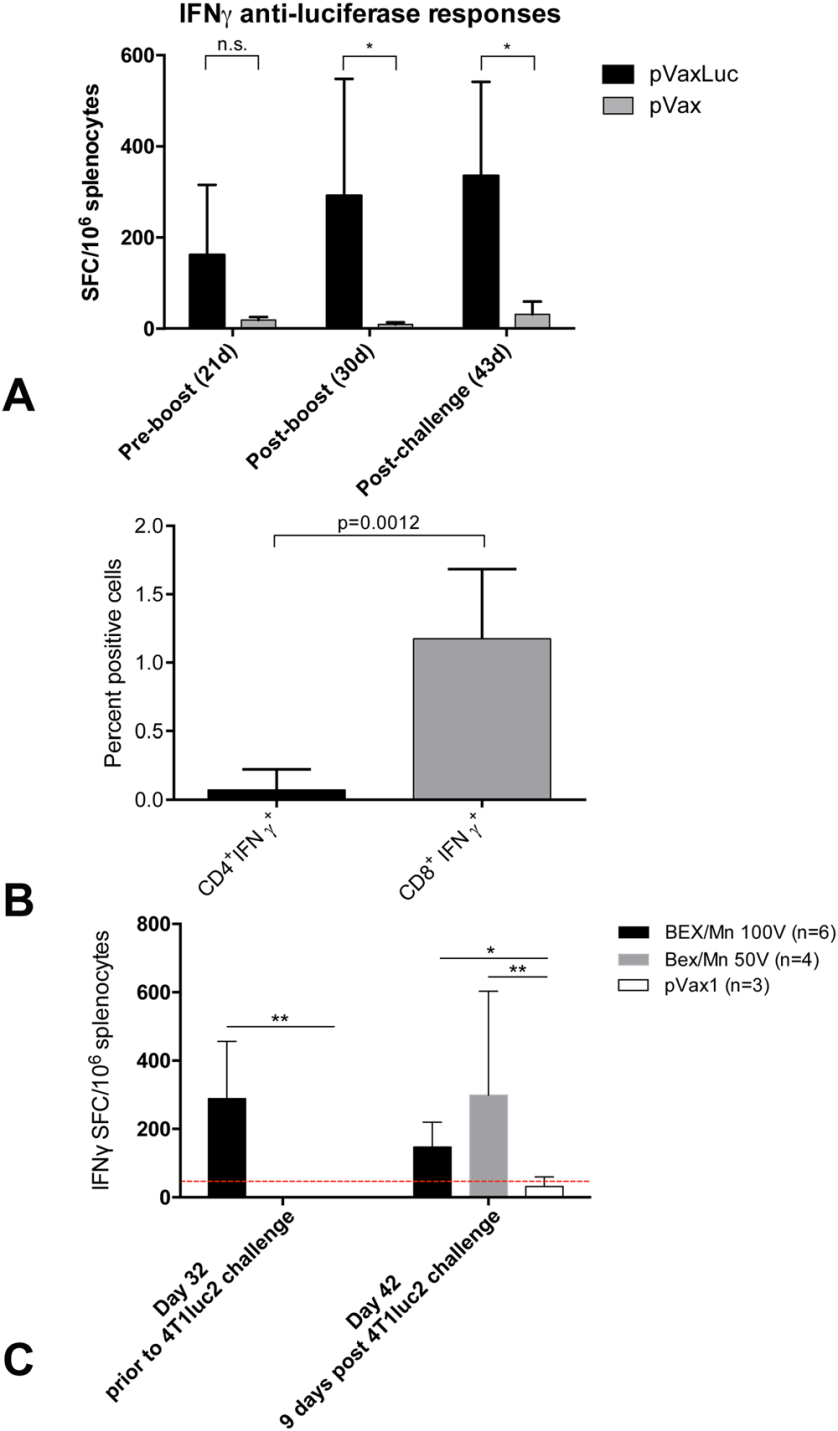
Cellular immune response to peptide GFQSMYTFV representing an immunodominant CTL epitope of luciferase (LucP; ref. 21) in mice immunized with Luc DNA (pVaxLuc). The average number of IFN-γ producing splenocytes per mln cells in mice receiving pVaxLuc followed by electroporation using Dermavax (n = 6), or BEX devices at 100 V (n = 6), or empty vector pVax1 with electroporation using BEX device at 100 V (n = 6) after priming (day 21), boosting (day 30), and after challenge with 4T1luc2 cells (day 9, or 43 from the start) (see Methods for details) (**A**); FACS of the pooled splenocytes of Luc-gene immunized mice detecting LucP-specific secretion of IFN-γ by CD8 + T cells (**B**); Average LucP-specific IFN-γ response of mice immunized with Luc DNA and electroporated using CUY21EditII (BEX) at 50 V and 100 V (**C**). Red line indicates the cut-off for specific IFN-γ response as number of spots registered in non-immune (control and vector immunized) mice + 3 STDV. *p < 0,05, Luc DNA immunized mice electroporated at 100 V, and **p < 0,1, Luc DNA immunized mice electroporated at 50 V compared to vector immunized mice (Mann-Whitney test; STATISTICA AXA 10.0).

Resistance to tumor growth coincided with the induction of an IFN-γ response to CD8 T-cell epitope of luciferase represented by LucP peptide^21, 22^, detectable after both prime and boost with Luc DNA (Fig. 9A). FACS with intracellular staining indicated that anti-LucP response was mediated mainly by CD8+ T cells (Fig. 9B).

LucP-specific IFN-γ response was induced after intradermal injections followed by electroporation with driving pulses of 100 V, whereas pulses of 50 V induced no response (Figs 9C and S4D). Interestingly, mice immunized with Luc DNA and electroporated at 50 V reacted to the implantation of 4T1luc2 cells by an anamnestic Luc-specific T-cell response, attributable to the antigen boost by Luc expressed by the growing 4T1luc2 cells (Figs 9C and S4E). No IFN-γ response to luciferase was registered in the non-immunized (naïve or control) mice up to 9 days post the 4T1luc2 challenge (Fig. 9A,C). No antibodies to Luc were detected in either 4T1luc2D6, or 4T1luc2-implanted, or pVaxLuc-immunized mice at any time point.

## Discussion

Genetic labelling of tumor cells with bioluminescent reporters allows detection of as few as three reporter-labelled cells residing subcutaneously within a mouse^12, 23^, supports high throughput screening and provides a relative measure of cell viability (as dead cells cannot support the enzymatic reaction and do not emit signal)^24, 25^. It was previously shown that BLI is perfectly suitable for monitoring the total tumor cell burden, detecting and quantifying circulating tumor cells, metastases, and evaluating cellular metabolism, as means of monitoring the efficacy of anti-tumor treatments^24, 26^. The volume and weight of the labeled tumor strongly correlate to the intensity of signals generated by the reporter-labelled cells enabling quantitative assessment of the tumor’s growth and metastatic activity^12, 27^. One of the most frequently used and best studied bioluminescent reporters is the firefly luciferase (Luc). Here, we have characterized the capacity of two Luc-labelled subclones of the murine mammary gland adenocarcinoma 4T1 cells, 4T1luc2D6^16^ and 4T1luc2 (Perkin Elmer), to generate tumors and metastases in the immunocompetent mice in comparison with the parental 4T1 cells. In lines with the earlier findings, we found that expression of the luciferase has no effect on either the *in vitro* proliferation of 4T1 cells, or on their ability to form tumors after the orthotopic transplantation^7, 9, 12, 18, 28^. However, when growing *in vivo*, both 4T1luc2D6 and 4T1luc2 cell lines significantly reduced the levels of reporter expression. Furthermore, the morphometric analysis showed that 4T1luc2D6 tumors were growing approximately 1.5 times slower than tumors formed by 4T1 cells. Thus, although two Luc-expressing 4T1 clones had *in vitro* proliferation rates equal to that of the parental 4T1 cells, and similar propensity to form primary tumors, *in vivo* they were growing slower (at least 4T1luc2D6) than the parental 4T1 clone. In the study by Tiffen J *et al*., tumors formed by GFP-P2A-luc cells expressing Luc were also growing significantly slower than tumors formed by the parental B16-F10 expressing only GFP^18^. A study by Brutkiewicz S *et al*. also revealed that tumor cell growth could be hampered by the expression of foreign proteins such as luciferase^29^.

We attributed the phenomenon to the anti-tumor immune response induced by the expression of reporter. Likewise, Tao *et al*. observed that 4T1 tumors established in normal BALB/c mice demonstrate initial (2 weeks post implantation) substantial loss of tumor cells; whereas no loss was observed in either BALB/c nude, or BALB/c SCID mice^11^. Temporal bioluminescence loss was attributed to the humoral immune response against 4T1-associated antigens^11^. Tumors re-emerged at the later stages, which was explained by an immune suppression resulting from the tumor-induced expansion of myeloid derived suppressor cells^11^. Our data reproduced the initial stage of this bi-phasic development (bioluminescence loss after day 15). Reporters such as luciferase and GFP are foreign antigens that can induce an immune response in immunocompetent animals^21, 22^. We have not observed any antibody response against Luc in mice bearing either 4T1uc2, or 4T1luc2D6 tumors. Therefore, we hypothesized that the impeded tumor growth could be caused by the suppression of 4T1luc-tumor growth by the T cell response against luciferase.

To prove if this is the case, we tested mice implanted with Luc-expressing 4T1 cells for the presence of Luc-specific immune response. By the experimental end-point, splenocytes of all mice with tumors reaching the area of 1.0 cm^2^ exhibited an IFN-γ response against the immunodominant CD8+ T cell epitope of luciferase recognized by BALB/c mice, registered by both IFN-γ ELISpot and flow cytometry, stronger in 4T1luc2D6, and weaker, in 4T1luc2-implanted mice (Fig. 7), but no specific antibodies. This confirmed the cellular immunogenicity of luciferase in the context of Luc-expressing tumors. We further tested 4T1luc2-implanted mice for the presence of Luc-specific immune response at different time points, from early (days 6 to 9) to the late stages of tumor development (day 23 after the implantation). Luc-specific cellular response was detected starting from day 9, coinciding with the slow-down in the rate of the tumor growth. These experiments confirmed that mice implanted with Luc-expressing tumors gradually develop T-cell response against the reporter.

Luc-specific immune response stimulates immune evasion, giving survival benefit to the tumor cells with reduced levels of luciferase expression. To check if this is the case, we compared the levels of Luc expression in the original cell lines, and in the primary cells isolated from 4T1luc 2 and 4T1luc2D6 tumors. We observed a considerable reduction of Luc expression compared to the implanted cell lines, 60-fold in cells derived from 4T1luc2D6, and 4-fold in cells derived from 4T1luc2 tumors (Fig. 3) illustrating the general concept of instability/variations in the epigenetic and gene expression landscapes of the tumors^30^.

The uniformity of the change in luciferase expression levels in tumors originating from the same mouse pointed at a common systemic mechanism of their downregulation. Reporter expressing tumor cells would be targeted by the immune response resulting in a T-cell mediated killing of cells with significant, while not affecting cells with negligible levels of reporter expression. This scenario is realized in case of the vector-directed *in vivo* transfer of the transgenic products for treatment of genetic and acquired diseases. Antigen-specific adaptive immune response reduces both the stability of transfer and the efficacy of transgene expression^30^. For example, in the experiments with sleeping beauty-mediated Luc gene transfer, continuous high-level expression of luciferase induced a strong CTL response, which resulted in a rapid loss of the transgene expression in the immunocompetent host^31^. In our case, 4T1luc2 and 4T1luc2D6 had similar growth characteristics, and similar levels of reporter expression *in vitro* (Fig. 1). Both were subjected to immune pressure after the implantation, generating a detectable anti-Luc IFN-γ response (Fig. 7); and both reduced reporter expression (Fig. 3). In case of 4T1luc2D6, anti-LucP response was stronger and resulted in a stronger suppression of Luc expression and in a significant reduction of tumor growth (Fig. 2).

In addition to the adaptive immune response, the loss of reporter signal can result from IFN-γ mediated down-regulation of Luc expression from viral promoters^32–34^. The 4T1luc2D6 clone was obtained by the stable transfection of 4T1 cells with plasmid DNA containing Luc2 gene controlled by the immediate early CMV promoter^16^ and 4T1luc2, by the transduction of 4T1 with a Luc2-encoding lentivirus^17^. The number of lentiviral copies/Luc gene inserts in 4T1luc2 cells approaches 500, and is at least 10-fold higher than in 4T1luc2D6 (qPCR, Isaguliants M, unpublished) with a high initial level of Luc expression in 4T1luc2D6 cells warranted by the strength of the CMV promoter^35^. Both CMV- and lentiviral promoters can be subjected to a non-cytolytic down-regulation of expression. The suppression of the CMV-driven expression from few plasmid inserts is more likely to occur than the downregulation of expression from the multiple lentiviral inserts^36^. This may explain significantly lower levels of reporter expression in cells derived from 4T1luc2D6 compared to 4T1luc2 tumors.

Clone 4T1luc2 appeared to be less susceptible than 4T1luc2D6 to both the immune-mediated suppression of tumor growth, and to the downregulation of reporter expression, supporting the use of viral transduction as a preferable method for generation of stable cell lines for cancer research. It may be argued that higher Luc expression levels should induce a stronger immune response against Luc, and a more efficient clearance. However, Luc expression levels in tumor cells showed no correlation with the immune response against the reporter. Rather, less malignant 4T1luc2D6 subclone induced a stronger anti-Luc immune response than more malignant 4T1luc2 subclone, irrespectively of the difference in Luc expression levels. An immune suppression induced by the tumors interferes with the immune control of primary tumor growth, specifically at the later stages of tumor development^11^, and may explain the lack of a sustained immune control of 4T1luc2 tumor growth. Susceptibility of tumors formed by reporter-labelled cell lines, specifically those generated by stable transfection of reporter gene(s), to the immune-mediated down-regulation of reporter expression, whichever are them mechanisms, limits their applications, since BLI measurements of these tumors have to be complemented by morphometry.

We have further inquired if the reporter expression affected the metastatic potential of Luc-expressing 4T1 clones. We characterized the metastatic activity of Luc-expressing 4T1 tumors using a combination of bioluminescent imaging (BLI), MRI, and micro-CT. The minimum size of metastases detectable by BLI was 0.3 × 0.5 mm for the thoracic organs and 0.2 × 0.2 mm for other tissues located close to the body surface (Figs 4, 5 and 6). Combination of three modalities reliably detected metastasis of the primary 4T1luc2D6 tumors in the regional lymph nodes and in the soft tissues of the neck, legs, lungs, liver, abdominal cavity, retroperitoneal fat, and skeletal bones. The metastases into skeletal bones (in particular, those in vertebral bodies) were detected by the targeted MRI and micro-CT only at the later stages, after the appearance of the corresponding clinical symptoms. Densitometry, highly sensitive in the case of the osteolytic metastases, could not detect them at the stage of development preceding bone resorption. However, they were reliably detected by BLI with MRI verification. Surgical removal of the primary focus improved the detection of metastases^24, 28^. It allowed the detection of a weak bioluminescence from the metastases located deep in tissue, and also activated the development of metastases, making the model more similar to metastasizing human BC (with primary focus removed in most cases). Removal of the primary focus revealed the development of microscopic 100 μm dormant metastasis formed by the 4T1luc2D6 tumor in the mouse brain, not captured by BLI, but confirmed by histochemical analysis(Supplementary Fig. S2). Analysis by the combination of methods revealed that mice implanted with the parental 4T1 cells exhibited 11.4 ± 2.1 metastases per mouse, with 4T1luc2D6 cells, 4.5 ± 0.6 (p < 0.01, Table 1). None of the methods detected metastases in the 4T1luc2 implanted mice. To confirm this, we transcardially perfused the euthanized mice with a buffered 4% paraformaldehyde solution and, after fixation, subjected their internal organs to the histological examination. A comparison of MRI and 3D reconstruction of BLI images with the histological data demonstrated that both visualization techniques gave only an approximate estimation of the number of metastases (due to the superposition of bioluminescent signals from the adjacent objects, as well as scattering of bioluminescence in the tissues). Histological examination revealed that BLI and MRI failed to detect brain metastases of the Luc-expressing 4T1 tumors if they were less than 100 μm in diameter (150 to 200 cells). After the histological examination, we were able to detect two dormant brain metastases sized 100 μm in mice implanted with 4T1luc2D6, and still none in the 4Tluc2-implanted animals.

This demonstrated that Luc-expressing 4T1 tumors had a restricted metastatic activity compared to the tumors formed by the parental 4T1 cells. Specifically, they generated fewer distal metastases (as those in the liver) and very few or no metastases in brain. The metastatic potential was significantly reduced for 4T1luc2D6, and abrogated for 4T1luc2 clone (at least in experiments with duration below 4 weeks). Indeed, stably high level of *in vivo* reporter expression observed for 4T1luc2 tumors (Fig. 3) affected their metastatic activity much more than it affected the primary tumor growth. The restricted metastatic activity of Luc-expressing 4T1 cells could be due to the inhibitory effects of luciferin oxidation on the mitochondrial activity and cell viability^37^, which would be more pronounced for tumors with higher levels of Luc expression (here, 4T1luc2 ones). The effect may also be attributed to the Luc-specific immune response against single migrating 4T1luc2 cells.

Strangely, data on the reduced invasiveness of genetically labeled tumor cells in the immunocompetent non-transgenic animals appeared to be very limited. Of the multiple Luc-labelled tumor models described so far, such as breast carcinomas, adenocarcinomas of colon^38^ and breast^12, 28, 39^, lymphomas^40^ or retinoblastomas^41^, none reported reduced invasiveness or diminished capacity to form metastases compared to the parental non-labelled tumors. Importantly, neither cell culture nor mouse xenograft assays showed any significant functional differences between the luciferase-transfected and parental cells, although single observations were made that a continuous exposure of cell cultures to luciferin may lead to impaired mitochondrial activity and restricted cell growth^37^. On this uneventful background, two studies registered a low number of distal metastases formed by 4T1-derived clones^36^. We and others have also shown that the expression of fluorescent reporters (as GFP, or near-infrared fluorescent proteins/iRFP) considerably reduces the invasiveness of tumor cells, and may lead to spontaneous tumor regression^16^, and even to inability to establish the primary tumor^16^ (as in case of iRFP, whereas same tumors could be easily implanted into mice transgenic for the given reporter)^42^.

In support of the concept of immune restriction of tumor growth and invasiveness, we immunized BALB/c mice with a plasmid encoding luciferase (Luc DNA). A successful immunization induced IFN-γ response to the immunodominant CTL epitope of luciferase, and protected mice from the establishment of 4T1luc2 tumors. This response was mediated mainly by CD8+ T-cells, corroborating the earlier findings on the capacity of Luc-specific CD8+ T cells to restrict tumor growth^14^. Interestingly, DNA-immunization employing electroporation at a lower voltage which promoted lower levels of luciferase expression, induced low or no T-cell response and conferred protection against the 4T1luc2 challenge to only one animal. Thus, the systemic immunity to reporter(s) expressed by the tumors, manifested by IFN-γ response to a CTL epitope, can suppress both the induction and growth of the primary tumors.

Can an immune response against the reporter explain the absence of metastases of primary 4T1luc2, but not of 4T1luc2D6 tumors? Micro-metastases may be formed very early, during the first week after the implantation of tumor cells^43, 44^. Studies in a mouse model of melanoma have also shown that the malignant cells are disseminating in the early stage of primary tumor development, even before it becomes detectable^45^. Not all micro-metastases would later turn into the actual metastases. For luciferase-expressing D3H1 and D3H2LN clones (derived from human metastatic ductal breast carcinoma cell line MDA-MB-231 and provide rapid mammary tumor growth) the outgrowth of the spontaneous distal metastases coincided with the increase in the volume of the primary tumor by week 4^46^, i.e. with the second immunosuppressive phase of tumor development^11^. At the early stage of tumor growth, before the induction of tumor-induced immunosuppression, an immune response against tumor-associated antigens (modelled here by luciferase) may limit the spread of 4T1 cells when they migrate as single cells in the vascular system. In lines with this, at an early stage of tumor development (two weeks after the implantation) GFP-expressing 4T1-derived tumors exhibited low metastatic activity generating fewer metastases in brain, bones or liver than the parental 4T1 tumors^47^. Thus, growth characteristics and metastatic activity of 4T1luc cells together with the data on the immunogenicity of Luc reporter in mice implanted with Luc-expressing 4T1 tumors demonstrate that the reporter-specific immune response can control and ultimately prevent metastasis formation during the first two weeks of tumor development. At least a part of the immune response against Luc is constituted by the CD8+ T cell response against its immunodominant CTL epitope. In a mouse model of melanoma, the early outgrowth of the metastases was also restricted to the CD8+ T cells, since CD8+ T cell depletion led to their rapid outgrowth^45^. All underlying mechanisms of immune control of metastasis formation are yet unclear^45, 48^, however the data obtained here in the orthotopic mouse model of breast cancer strongly supports the importance of the CTL-mediated control of metastatic activity. Possible way to evade the immune interference for the luciferase-labelled tumor cells and broaden the applicability of the model would be to decrease immunogenicity of the reporter by deleting or modifying its major CTL epitopes.

Our results also point at the options this and similar animal models based on the genetically labelled tumor cells offer for cancer vaccine development. Earlier, it was shown that Luc DNA immunization can induce a CTL-mediated resistance to the growth of Luc-expressing tumors^14^. In case of 4T1 tumors, an effective immune response against one of the tumor associated antigens, fibroblast activation protein α overexpressed by cancer-associated fibroblasts, successfully reduced 4T1 tumor growth, also in a CTL-mediated fashion^49^. Our data on unsuccessful Luc DNA-immunization reveals that cancer vaccine candidates can be easily compromised by an inappropriate administration mode. In this context, DNA-immunization with the reporter genes followed by challenge with the reporter-expressing tumor cells, presents a unique opportunity to optimize immunogen delivery, compare different DNA-immunization regimens, and further optimize to induce anti-tumor immune response with strong lytic capacity. Recently, 4T1model was used to reveal a “window of opportunity” for the therapy/immunotherapy of cancer which opens after the resection of primary tumors, as 10 days characterized by the decreased tumor-associated immune suppression^50^. Reporter expressing 4T1 cells would offer a possibility to test this as an opening for anti-cancer immunotherapy.

## Conclusions

In summary, we have quantitatively characterized the invasiveness and metastatic activity of the murine breast carcinomas formed by two 4T1-based cell lines genetically labelled with luciferase. Combination of optical imaging, MRI and micro-CT enabled us to evaluate the rate of tumor growth and reliably assess the potential of both cell lines to form metastasis in various tissues and organs. The study demonstrated an aberrant metastatic potential for both, and a reduced invasiveness for one of the of Luc-expressing 4T1 cell lines. We attributed the restrictions to an immune response induced against luciferase. An immune response to a CD8+ T cell epitope of luciferase was detected in BALB/c mice implanted with Luc-expressing tumors, and was stronger in mice that were able to restrict tumor growth. Furthermore, both Luc-expressing cell lines had reduced metastatic activity compared to the parental 4T1 clone, more pronounced for 4T1luc2 clone characterized by stably high level of *in vivo* reporter expression. Immunogenicity of luciferase may compromise the metastatic activity of the reporter-expressing tumor cells, a drawback to be considered when working with this and similar models. We also used 4T1luc2 cells for optimization of DNA-immunization regiments exploiting luciferase as a tumor-associated antigen. In these experiments, we demonstrated that the use of the optimal electroporation protocol promoted an immune response with protective potential, whereas suboptimal regimens inducing weak response were unable to prevent the establishment of primary tumors. Thus, the model of reporter-expressing tumors offers a powerful instrument for improving the DNA-immunization technique, and is highly valuable for the development of efficient cancer vaccines/vaccination regiments.

## Methods

### Cell lines

4T1^51^ (CRL-2539™, ATCC) and 4T1luc2 (BW124087, Perkin Elmer, Waltham, MA) were purchased; 4T1luc-D6 clone was described previously^16^. Cells were cultured and passaged as recommended by the manufacturers. Both cell lines express luciferase encoded by luc2 gene (http://www.perkinelmer.com.cn/CMSResources/Images/46-176379PST-WMIC2015-Using-brighter-red-shifted-luciferase-*in-vivo*-tumor-bioluminescence-imaging.pdf) Luc2 is a synthetic gene for “yellow” firefly luciferase engineered to improve mammalian expression through codon optimization (Promega; https://se.promega.com/products/reporter-assays-and-transfection/reporter-vectors-and-cell-lines/pgl4-luciferase-reporter-vectors/promoterless-firefly-luciferase-vectors/). The time course of the proliferation of the parental 4T1, and 4T1luc2D6 and 4T1luc2 (PerkinElmer) clones was determined using Nikon Biostation CT (Nikon, Japan).

### Animals

Eight-week old female BALB/c mice were purchased from Charles River (Germany) or Andreevka Nursery (tBALB/cfC3H; Russia). Animals were housed 6–10 per cage under a light-dark (12 h/12 h) cycle with ad libitum access to water and food. Experimental manipulations were performed under the inhalation anesthesia induced by 4% and maintained by 2.3% mixture of isofluorane in oxygen administered through facial masks. Minor interventions were done under local anesthesia (2% lidocaine). Experiments were carried in compliance with the bioethical principles adopted by the European Convention for the Protection of Vertebrate Animals Used for Experimental and Other Scientific Purposes (Strasbourg, 1986), Order of the Ministry of Health of the Russian Federation of August 23, 2010 “Establishment of the Rules of Laboratory Practice” No. 708n. The experimental protocol was approved by Local Ethical Committee of Federal Research and Clinical Center of Specialized Medical Care and Medical Technologies FMBA of Russia (No10a from 09/12/2015), and Ethical Committee for Animal Experiments of the North Stockholm region N66_13.

### Luciferase expression

By 4T1lucD6 and 4T1luc2 clones was evaluated *in vitro* and *in vivo*. *In vitro* measurements were performed in cell culture fluid of Luc-expressing 4T1 cells grown in a 96-well cell culture plate. Growth medium was removed and wells were filled with 100 μl luciferin solution (150 μg/ml; Perkin Elmer) in the complete RPMI containing 2 mM L-glutamine, 2 mM Penicillin-Streptomycin (Sigma-Aldrich) and 10% FBS (Gibco, Invitrogen, Carlsbad, CA). The chemiluminescence intensity after a 5-min exposure was measured on an EnSpire reader (Perkin Elmer). Comparison of the level of Luc2 expression by 4T1lucD6 and 4T1luc2 was done by growing them in the same conditions and registering the chemiluminescence on Spectrum CT using limiting cell dilutions. The number of photons/cell/sec was calculated based on the total photon flux and cell numbers. Comparison of the level of Luc2 expression by primary cells derived from 4T1lucD6 and 4T1luc2 tumors was carried out on multiplate reader Enspire (Perkin Elmer). Luminescence intensity measured by Enspire reader correlates with luminescence intensity obtained on IVIS Spectrum CT (see Supplementary Fig. 1).

### Implantation of 4T1 cells

Subclones of 4T1 cells grown in the selective medium were detached, sedimented, washed with serum-free RPMI-1640, stained for viability with Trypan Blue dye (Life Technologies, Carlsbad, CA), then counted in a hemocytometer, and aliquoted 5 × 10^2^ to 5 × 10^5^ cells in 50 μl of RPMI-1640 in sterile test tubes. Mice (n = 20) were orthotopically implanted with a suspension of either 4T1, or 4T1luc2D6, or 4T1luc2 cells, administered into the fat pad of the third pair of mammary glands using insulin syringes with 32 G needles. Bioluminescence imaging (Spectrum CT), X-ray micro-CT and *in vivo* MRI (ClinScan, Bruker BioSpin, Germany) were performed on days 1, 7, 14, 21, 28. Mice were sacrificed if the tumor size reached 1000 mm^3^. Additional groups of mice with 4T1, 4T1luc2D6 or 4T1luc2 tumors (n = 10) underwent a radical resection of the primary focus 21 days after implantation, and were scrutinized for metastasis by regular MRI and/or BLI examinations.

### X-ray and optical tomography

were carried on Spectrum CT according to the manufacturer’s protocols. The medium-resolution mode (FOV = 6 × 6 × 3 cm (l × w × h), voxel size = 75 µm) was used for X-ray micro-CT of most parts of the body; some body parts (knee joints, spine, and pelvic bones for X-ray densitometry) were scanned using the high-resolution mode (FOV = 2.4 × 2.4 × 2 cm (l × w × h), voxel size = 40 µm). Ten min prior to BLI, mice were intraperitoneally injected (150 mg/kg) with a 30 mg/ml solution of D-luciferin (Promega, or Perkin Elmer) in PBS. Bioluminescence was detected in the automated exposure mode. The 3D-reconstruction of the bioluminescence signal and its alignment with the micro-CT data were performed using Living Image 4.4 software (Perkin Elmer).

### MRI examination

was carried using ClinScan scanner (Bruker BioSpin) with an induced magnetic field of 7 T. The images were obtained using a 20-cm volumetric coil as a transmitter and a two-segment surface coil as a receiver of the RF signal. Mice were scanned in three stages: the posterior (posterior thoracic, lumbar, and pelvic regions), the anterior (cervical and thoracic regions) parts of the body, and the head. First, fast orthogonal T1-weighted images were obtained for spatial location; then, fat-suppressed T2-weighted turbo spin–echo (TSE) images were made in the coronary and sagittal planes for the anterior and posterior parts of the body and in the transversal plane for the head. In T2-weighted imaging, the voxel sizes were 0.156 × 0.156 × 1.0 mm for the trunk and legs and 0.123 × 0.104 × 0.6 mm for the head; the total duration of the examination of each mouse was 30 min.

### Volumetry of the primary tumor focus

Tumor growth was monitored by external caliper measurements (tumor size = [length × width × height] × 0.52), T2-weighted MRI, and for Luc-expressing tumors, by bioluminescent imaging. The most reliable was the determination of the volume of primary focus from the scans of the T2-weighted MRI. Tumors were mostly of round shape, allowing to determine the volume from the equation for ellipsoid (*V* = 4/3*πabc*, where *a*, *b*, and *с* are the ellipsoid semiaxis lengths). The semiaxis lengths were selected in the scan where the tumor was the largest. For tumor of irregular shape, volumes were determined from T2-weighted images using ImageJ software (NIH, Bethesda, MD). First, the area of the tumor was measured in each individual section; then, the tumor’s total volume was calculated as *V* = (*S*1+ … +*S*n)×(*h* + *d*), where *V* is the volume; *S*1 … n, area measured in each section; *h*, section thickness, and *d*, interval between sections. After that, volume values calculated for the coronary and transversal orthogonal planes were averaged. Estimations obtained using the ellipsoid volume equation and the summation of the areas in all scans differed by less than 15%.

### Quantification of the number of Luc-expressing 4T1 cells *in vivo*

Mice were subcutaneously injected with 10^2^ to 10^5^ 4T1luc2 cells, and imaged directly after the implantation of tumor cells. Measurements were performed in two to four repeats. Calibration curves were built, relating the number of injected cells to the total photon flux from the area of injection.

### Metastasis detection and verification

All tumor implantation experiments were performed on groups of at least five mice. In a standard experiment, mice (n = 20) were orthotopically implanted with the suspension of 4T1, 4T1luc2 or 4T1luc cells. Metastases formed by 4T1, 4T1luc2D6 and 4T1luc2 tumors were assessed by *in vivo* MRI (ClinScan, Bruker BioSpin, Germany) performed every 2–3 days after tumor implantation. MRI procedure and quantification of MRI data are described in detail in the Materials and Methods section. Metastasis of Luc-expressing tumors were also assessed by BLI (Spectrum CT, Perkin Elmer). In one experimental series done on mice implanted with 4T1 and 4Tluc2D6 tumors (n = 10/group) animals were additionally subjected to X-ray micro-CT with the medium, or high resolution modes (FOV = 6 × 6 × 3 cm/l × w × h; voxel size 75 μm; or FOV = 2,4 × 2,4 × 2 cm/l × w × h; voxel size 40 μm, respectively). Additional groups of five mice per implanted cell type were injected with a suspension of either 4T1, or 4T1luc2D6, or 4T1luc2 cells. After three weeks, mice underwent a radical resection of the primary focus, and were thereafter followed weekly by MRI and BLI (except mice with imlanted 4T1 cells) to detect the metastasis. Metastasis detected by micro-CT, and or 3D BLI after the removal of the primary focus were verified by histochemisty with hematoxylin-eosin staining of sections made from the paraffin-block preparations using protocols described for mouse studies^52^.

### Immunization of mice with Luc-encoding plasmid and subsequent challenge with 4T1luc2 cells

Immunization was made with plasmid pVaxLuc encoding “yellow” firefly luciferase^53^. Female BALB/c mice (9 week-old; 12 per group) were primed and four weeks later boosted with two intradermal injections of 20 μl PBS containing 20 μg pVaxLuc2 (kindly provided by Dr Maltais AK, Sweden), or pVax1 (Thermo Fisher Scientific, Waltham, MA) administered at the base of the tail with 29 G insulin syringes. Injections were followed by electroporation with needle-array electrodes (BTX, #47–0040) connected to DermaVax (Cellectis, France; ref. 22) or CUY21EditII electroporators (BEX Ltd, Japan). The latter employed the poration pulse of 400 V followed by eight driving 10-ms pulses of constant polarity at 50 V or 100 V delivered with 20-ms gaps. Two weeks after prime, mice were bled to assess the immune responses of peripheral blood mononuclear cells (PBMCs; below). Two weeks after the boost, six mice per group were sacrificed and their spleens were collected for immune assays. The remaining mice were challenged with 5000 4T1luc2 cells suspended in 50 μl of serum-free DMEM (HyClone), injected subcutaneously at two sites on the back above the immunization sites. Mice were monitored by BLI directly after the implantation, and then regularly until the experimental end-point manifested by the establishment of solid tumors in all animals of the control group (day 43/9 days after implantation).

### Analysis of cellular response to luciferase in peripheral blood mononuclear cells and splenocytes by ELISPOT

On days 20, 30 to 34, mice were bled, blood was pooled per group, treated with RBC-lysis buffer to remove the thrombocytes, and PBMC were collected by centrifugation. On day 43, mice were sacrificed, spleens were harvested, homogenized, and splenocytes were isolated by gradient centrifugation in Ficoll-Plaque Plus (GE Healthcare Amersham Biosciences, USA). Pooled PBMCs or individual mouse splenocytes were distributed 10^5^/well and stimulated for 20 hours with the complete RPMI containing 6 to 12 μg/ml recombinant luciferase (Promega), or 10 μg/ml of luciferase-derived peptide GFQSMYTFV^54^ (GL Biochemical, Shanghai, China), or Concanavalin A (ConA; 5 μg/ml; Sigma), or RPMI alone. Stimulations were performed in duplicates. IFN-γ production by stimulated lymphocytes was assessed 20 h later using IFN-γ ELISPOT kit (Mabech, Nacka, Sweden) as recommended by the manufacturer. ELLLISPOT plates were read on the iSpot reader (AID GmbH, Strassberg, Germany).

### Analysis of cellular response to luciferase in splenocytes by flow cytometry with intracellular cytokine staining

All reagents used for FACS analyses were purchased from BD Biosciences (Franklin Lakes, NJ, US) unless stated otherwise. Splenocytes of immunized or control mice (3 × 10^6^) were stimulated for 4–6 hr at 37 °C and 5% CO_2_ with GFQSMYTFV (10 μg/ml) or RPMI alone, or ConA as a positive control. The stimuli were diluted in RPMI supplemented with 5% FBS, 100 U/ml penicillin, 100 μg/mL streptomycin, and 0.3 mg/ml glutamine (Gibco) and GolgiPlug. To block unspecific binding of immunoglobulins to Fcγ receptors a CD16/CD32, antibody was added to each well 10 minutes before the end of the incubation. Before proceeding to staining surface molecules, cells were stained for viability using the Fixable Viability Stain 660 (FSV660) as recommended by the manufacturer. Surface staining was then performed by incubating the cells with a mixture of antibodies including: FITC-conjugated anti-mouse CD8a, APC-H7-conjugated anti-mouse CD4 and PerCP-conjugated anti-mouse CD3. Thereafter, cells were fixed and permeabilized for 20 minutes in 100 μl Cytofix/Cytoperm solution, washed with Perm/Wash buffer, and stained at 4 °C with PE-Cy7-conjugated anti-mouse IFN-γ antibodies. Stained samples were analyzed on a FACSVerse cytometer (BD Biosciences). Data were exported as FCS3.0 files using the FACSuite software. The FCS files were read using BioConductor’s^55^ package flowCore^56^ in the R software language. Finally, the cytometry data were normalized using the flowStats package^57^ and gated. First, a general lymphocyte area was defined and viable cells were identified by the lack of FSV660 staining. From the viable population, single cells were defined by the expression of surface markers and IFN-γ production.

### Analysis of humoral immune response

Blood was collected from all mice prior to the start of immunization, and on days 20, 30 to 34 and 43. Intermediate blood samples were drawn from the tail vein, and samples at the end-point of the experiment, by cardiac puncture. Serum samples were obtained and frozen at −20 °C until the analysis. ELISA was performed for all samples simultaneously at the end of the immunization/challenge experiments as was described by us previously^57, 58^.

## Electronic supplementary material

Supplementary info

## References

[1] Donepudi MS, Kondapalli K, Amos SJ, Venkanteshan P (2014). Breast cancer statistics and markers. J Cancer Res Ther..

[2] Weigelt B, Peterse JL, van’t Veer LJ (2005). Breast cancer metastasis: Markers and models. Nat Rev Cancer.

[3] Kiely D (2014). Timeliness in breast cancer care as an indicator of quality. Clin J Oncol Nurs..

[4] Lelekakis M (1999). A novel orthotopic model of breast cancer metastasis to bone. Clin Exp Metastasis..

[5] Coleman RE, Smith P, Rubens RD (1998). Clinical course and prognostic factors following bone recurrence from breast cancer. Br J Cancer.

[6] Kennecke H (2010). Metastatic Behavior of Breast Cancer Subtypes. J Clin Oncol..

[7] Khanna C, Hunter K (2005). Modeling metastasis *in vivo*. Carcinogenesis..

[8] Fantozzi A, Christofori G (2006). Mouse models of breast cancer metastasis. Breast Cancer Res..

[9] Bolin C, Sutherland C, Tawara K, Moselhy J, Jorcyk CL (2012). Novel mouse mammary cell lines for *in vivo* bioluminescence imaging (BLI) of bone metastasis. Biol Proced Online..

[10] Aslakson CJ, Miller FR (1992). Selective events in the metastatic process defined by analysis of the sequential dissemination of subpopulations of a mouse mammary tumour. Cancer Res..

[11] Tao K, Fang M, Alroy J, Sahagian GG (2008). Imagable 4T1 model for the study of late stage breast cancer. BMC Cancer..

[12] Kim JB (2010). Non-invasive detection of a small number of bioluminescent cancer cells *in vivo*. PLoS One..

[13] Sasportas LS, Hori SS, Pratx G, Gambhir SS (2014). Detection and quantitation of circulating tumor cell dynamics by bioluminescence imaging in an orthotopic mammary carcinoma model. PLoS One..

[14] Jeon YH (2007). Immune response to firefly luciferase as a naked DNA. Cancer Biol Ther..

[15] Day CP (2014). Glowing head” mice: a genetic tool enabling reliable preclinical image-based evaluation of cancers in immunocompetent allografts. PLoS One..

[16] Baklaushev VP (2015). Modeling and integral X-ray, optical, and MRI visualization of multiorgan metastases of orthotopic 4T1 breast carcinoma in BALB/c mice. Bull Exp Biol Med..

[17] Kim J-B (2010). Non-Invasive Detection of a Small Number of Bioluminescent Cancer Cells *In Vivo*. PLoS ONE.

[18] Tiffen JC, Bailey CG, Ng C, Rasko JE, Holst J (2010). Luciferase expression and bioluminescence does not affect tumor cell growth *in vitro* or *in vivo*. Mol Cancer..

[19] Conti E, Franks NP, Brick P (1996). Crystal structure of firefly luciferase throws light on a superfamily of adenylate-forming enzymes. Structure..

[20] Demicheli R, Retsky MW, Hrushesky WJM, Baum M, Gukas ID (2008). The effects of surgery on tumor growth: a century of investigations. Ann Oncol.

[21] Limberis MP, Bell CL, Wilson JM (2009). Identification of the murine firefly luciferase-specific CD8 T-cell epitopes. Gene Ther..

[22] Petkov SP (2013). Evaluation of immunogen delivery by DNA immunization using non-invasive bioluminescence imaging. Hum Vaccin Immunother..

[23] Rabinovich BA (2008). Visualizing fewer than 10 mouse T cells with an enhanced firefly luciferase in immunocompetent mouse models of cancer. Proc Natl Acad Sci USA.

[24] Lyons SK, Patrick PS, Brindle KM (2013). Imaging mouse cancer models *in vivo* using reporter transgenes. Cold Spring Harb Protoc..

[25] Dubey P (2012). Reporter gene imaging of immune responses to cancer: progress and challenges. Theranostics..

[26] Penet MF (2010). Applications of molecular MRI and optical imaging in cancer. Future Med Chem..

[27] Christensen J, Vonwil D, Shastri VP (2015). Non-Invasive *In Vivo* Imaging and Quantification of Tumor Growth and Metastasis in Rats Using Cells Expressing Far-Red Fluorescence Protein. PLoS One..

[28] Adiseshaiah, P. P. *et al*. Longitudinal imaging of cancer cell metastases in two preclinical models: a correlation of noninvasive imaging to histopathology. Int J Mol Imaging. 102702, doi:10.1155 (2014). 10.1155/2014/102702 PMC3958723 24724022

[29] Brutkiewicz S (2007). The expression level of luciferase within tumour cells can alter tumour growth upon *in vivo* bioluminescence imaging. Luminescence..

[30] Caiado F, Silva-Santos B (2016). Intra-tumour heterogeneity – going beyond genetics. FEBS.

[31] Podetz-Pedersen KM, Vezys V, Somia NV, Russell SJ, McIvor RS (2014). Cellular immune response against firefly luciferase after sleeping beauty-mediated gene transfer *in vivo*. Hum Gene Ther..

[32] Ghazizadeh, S., Carroll, J. M. & Taichman, L. B. Repression of Retrovirus-Mediated Transgene Expression by Interferons: Implications for Gene Therapy. *J. Virol* 9163–9169 (1997). 10.1128/jvi.71.12.9163-9169.1997 PMC230218 9371574

[33] Griffin DE (2010). Recovery from viral encephalomyelitis: immune-mediated noncytolytic virus clearance from neurons. Immunol Res..

[34] Dag F (2014). Reversible silencing of cytomegalovirus genomes by type I interferon governs virus latency. PLoS Pathog..

[35] Schlabach MR, Hu JK, Li M, Elledge SJ (2010). Synthetic design of strong promoters. PNAS.

[36] Shearer RF, Saunders DN (2015). Experimental design for stable genetic manipulation in mammalian cell lines: lentivirus and alternatives. Genes Cells..

[37] Feys, L. *et al*. Quantitative and Functional Requirements for Bioluminescent Cancer Models. *In Vivo*. 01-02;30(1), 1–11 (2016). 26709122

[38] Terracina KP (2015). Development of a metastatic murine colon cancer model. J Surg Res..

[39] Shibata MA (2009). An immunocompetent murine model of metastatic mammary cancer accessible to bioluminescence imaging. Anticancer Res..

[40] Edinger M (2003). Revealing lymphoma growth and the efficacy of immune cell therapies using *in vivo* bioluminescence imaging. Blood..

[41] Ji X (2009). Noninvasive visualization of retinoblastoma growth and metastasis via bioluminescence imaging. Invest Ophthalmol Vis Sci..

[42] Tran MT (2014). *In vivo* image analysis using iRFP transgenic mice. Exp Anim..

[43] Culp LA, Lin W, Kleinman NR, O’Connor KL, Lechner R (1998). Earliest steps in primary tumor formation and micrometastasis resolved with histochemical markers of gene-tagged tumor cells. J Histochem Cytochem..

[44] Culp LA, Holleran JL, Miller CJ (2001). Tracking prostate carcinoma micrometastasis to multiple organs using histochemical marker genes and novel cell systems. Histol Histopathol..

[45] Eyles J (2010). Tumor cells disseminate early, but immunosurveillance limits metastatic outgrowth, in a mouse model of melanoma. J Clin Invest..

[46] Jenkins DE, Hornig YS, Oei Y, Dusich J, Purchio T (2005). Bioluminescent human breast cancer cell lines that permit rapid and sensitive *in vivo* detection of mammary tumors and multiple metastases in immune deficient mice. Breast Cancer Res..

[47] Bailey-Downs, L. C. *et al*. Development and Characterization of a Preclinical Model of Breast Cancer Lung Micrometastatic to Macrometastatic Progression. *PLoS One*. 30, 9(**5**), e98624 (2014). 10.1371/journal.pone.0098624 PMC4039511 24878664

[48] Piérard-Franchimont C, Hermanns-Lê T, Delvenne P, Piérard GE (2014). Dormancy of growth-stunted malignant melanoma: sustainable and smoldering patterns. Oncol Rev..

[49] Xia Q (2016). Anti-tumor effects of DNA vaccine targeting human fibroblast activation protein α by producing specific immune responses and altering tumor microenvironment in the 4T1 murine breast cancer model. Cancer Immunol Immunother..

[50] Ghochikyan A (2014). Primary 4T1 tumor resection provides critical “window of opportunity” for immunotherapy. Clin Exp Metastasis..

[51] Pulaski BA, Ostrand-Rosenberg S (2001). Mouse 4T1 breast tumor model. Curr Protoc Immunol..

[52] Scudamor, C. L. Acquiring, Recording, and Analyzing Pathology Data from Experimental Mice: An Overview, “Current Protocols in Mouse Biology” Provider: John Wiley & Sons, Ltd, doi:10.1002/9780470942390.mo130200. 10.1002/9780470942390.mo130200 25715673

[53] Roos AK (2009). Skin electroporation: effects on transgene expression, DNA persistence and local tissue environment. PLoS One..

[54] Limberis MP, Bell CL, Wilson JM (2009). Identification of the murine firefly luciferase-specific CD8 T-cell epitopes. Gene Ther..

[55] Gentleman RC (2004). Bioconductor: open software development for computational biology and bioinformatics. Genome Biol..

[56] Hahne F (2009). flowCore: a Bioconductor package for high throughput flow cytometry. BMC Bioinformatics..

[57] Hahne F (2010). Per-channel basis normalization methods for flow cytometry data. Cytometry A..

[58] Starodubova E (2010). Potent cross-reactive immune response against the wild-type and drug-resistant forms of HIV reverse transcriptase after the chimeric gene immunization. Vaccine..

