# Supplementary material for: Luciferase Expression Allows Bioluminescence Imaging But Imposes Limitations on the Orthotopic Mouse (4T1) Model of Breast Cancer
Source: Sci Rep. 2017 Aug 10;7:7715. doi: 10.1038/s41598-017-07851-z (PMC5552689; doi:10.1038/s41598-017-07851-z)
Supplement: Supplementary file 1 — Supplementary info [file 41598_2017_7851_MOESM1_ESM.pdf]

LUCIFERASE EXPRESSION ALLOWS BIOLUMINESCENCE IMAGING BUT IMPOSES LIMITATIONS ON THE ORTHOTOPIC MOUSE (4T1) MODEL OF BREAST CANCER

Baklaushev VP[1,2]*, Kilpeläinen A[1,3], Petkov S[3], Abakumov MA[1], Grinenko NF[1], Yusubalieva GM[4], Latanova A A[5,6], Gubskiy IL[1], Zabozlaev FG[2], Starodubova ES[5,6], Abakumova TO[4], Isaguliants MG[3,6,7,8]*[#], Chekhonin VP[1,4#]

[1]Research and Education Center for Medical Nanobiotechnology, Pirogov Russian National Research Medical University, Ministry of Health of the Russian Federation; [2]Federal Research and Clinical Center of Specialized Medical Care and Medical Technologies, Federal Biomedical Agency of the Russian Federation, Moscow, Russia; [3]Department of Microbiology, Tumor and Cell Biology, Karolinska Institutet, Stockholm, Sweden; [4]Department of Fundamental and Applied Neurobiology, Serbsky National Research Center for Social and Forensic Psychiatry, Ministry of Health of the Russian Federation, Moscow, Russia; [5]Engelhardt Institute of Molecular Biology, Russian Academy of Sciences, Moscow, Russia; [6] Chumakov Federal Scientific Center for Research and Development of Immunobiological Preparations, Moscow, Russia; [7] N.F. Gamaleya Research Center of Epidemiology and Microbiology, Moscow, Russia; [8]Riga Stradins University, Riga, Latvia.

**Corresponding authors:*

Vladimir Baklaushev, MD,PhD, Pirogov Russian National Research Medical University, and Federal Research and Clinical Center of Specialized Medical Care and Medical Technologies, 28 Orekhovyi bulvar, 115682 Moscow, Russia; serpoff@gmail.com;

Maria Isaguliants, Associate Prof, PhD; N.F. Gamaleya Research Center of Epidemiology and Microbiology and Chumakov Federal Scientific Center for Research and Development of Immunobiological Preparations, 123098 Moscow, Russia; and Department of Research, Riga Stradins University, Dzirciema iela 16, LV-1007, Riga, Latvia maria.issagouliantis@rsu.lv

[#] *Shared last authorship.*

Minutes	Discovery?	P value	Mean1	Mean2	Difference	SE of difference	t ratio	df	q value
75	No	0.277116	21348	19600	1748	1521	1.149	10	0.391842
255	No	0.26257	24217	22698	1519	1280	1.187	10	0.391842
435	No	0.978091	26922	26945	-23.04	818.3	0.02816	10	0.942968
615	No	0.095755	31038	29883	1155	628.1	1.839	10	0.169247
795	Yes	0.00016	35726	30633	5092	869.5	5.856	10	0.003398
975	No	0.037506	37606	35485	2121	884.8	2.397	10	0.08839
1155	No	0.01838	43288	39580	3708	1318	2.813	10	0.064973
1335	No	0.2974	44133	45100	-967.3	879.9	1.099	10	0.394241
1515	No	0.003182	45877	48669	-2792	724	3.856	10	0.022493
1695	No	0.779784	50133	49804	328.5	1144	0.2873	10	0.787582
1875	No	0.02942	62512	55869	6643	2616	2.539	10	0.077999
2055	No	0.004731	72895	66825	6069	1679	3.615	10	0.025088
2235	No	0.43477	84189	81954	2235	2747	0.8137	10	0.512304
2415	No	0.021903	89841	94537	-4696	1732	2.711	10	0.066368
2595	No	0.001059	100725	111050	-10325	2269	4.55	10	0.011229
2775	No	0.517941	122691	125580	-2889	4311	0.6701	10	0.578185
2955	No	0.658367	148107	145954	2153	4725	0.4557	10	0.698198
3135	No	0.375986	170646	167147	3499	3777	0.9265	10	0.469097
3315	No	0.092055	182451	190127	-7677	4120	1.863	10	0.169247
3495	No	0.141079	213559	207145	6414	4013	1.598	10	0.230175
3675	No	0.077327	239627	229007	10620	5395	1.969	10	0.16401
3855	No	0.013446	278560	254667	23894	7977	2.996	10	0.05704

Supplementary Table. 1. Results of multiple t tests results comparing growth curves of 4T1Luc2 and 4T1Luc2D6 cell lines *in vitro* at all 22 time points. Statistical significance was determined using the Two-stage linear step-up procedure of Benjamini, Krieger and Yekutieli, with Q = 1%. Each test was analyzed individually, without assuming a consistent SD.

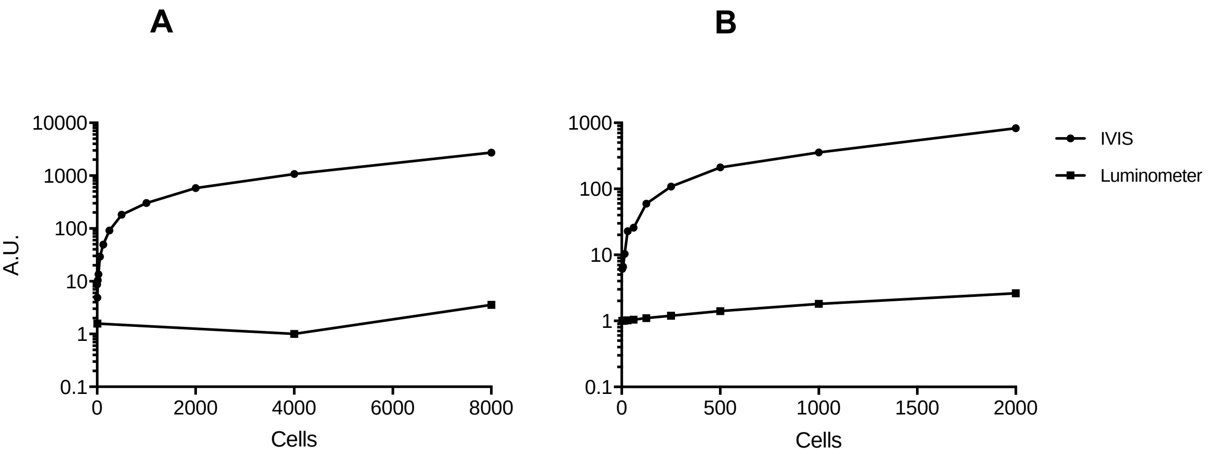

Supplementary Fig. 1. Correlation between the luminescence signal of 4T1lucD6 (A) and 4T1luc2 (B) cells registered by Spectrum CT (IVIS) and by Enspire devices (Luminometer). Pearson correlation coefficient, *r* between A.U. (photons/s) and cells was equal to 0.84 for 4T1lucD6, and 0.99 for 4T1luc2 cell lines.

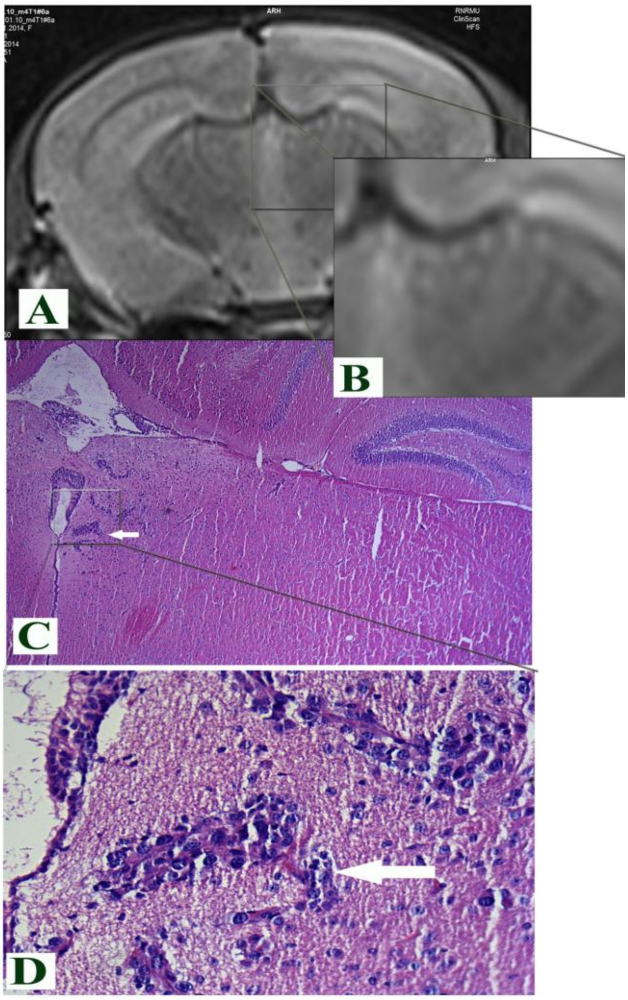

Supplementary Fig. 2 Visualization of brain metastases in mice implanted with 4T1 cells. Coronary projections by T2-weighted MRI image of the brain of a 4T1-implanted mouse 14 days after removal of the primary focus (A); magnified fragment of the MRI image, no metastases are clearly detectable (B); small perivasal metastases (less than 100 μm in diameter; indicated by arrow) in the paraventricular region of the thalamus, detected on a paraffin slice of the brain stained with hematoxylin–eosin (magnification 50×) (C); magnified fragment of brain slice, perivasal metastases indicated by arrow (magnification 200×) (D).

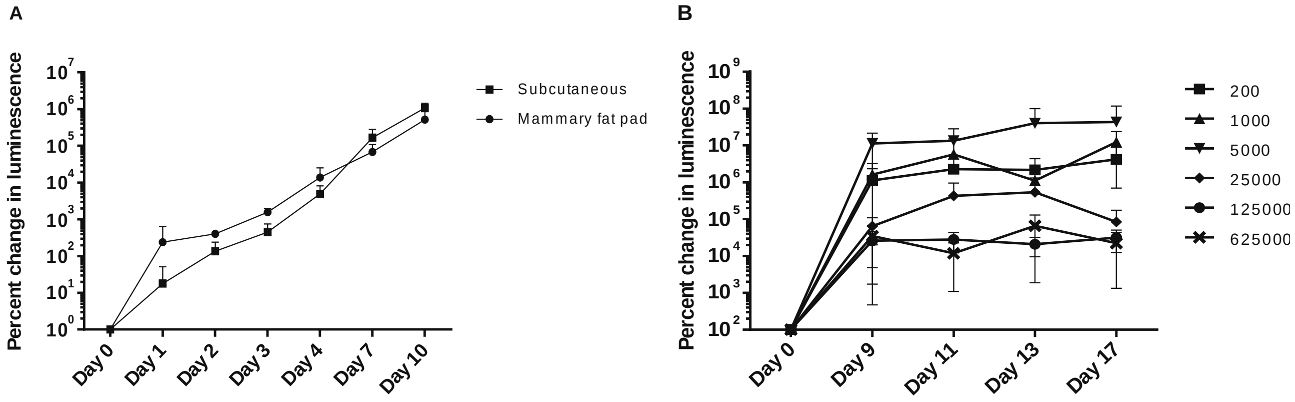

Supplementary Fig. 3 Conditions for implantation of 4T1luc2 cells allowing reliable early detection of the effects of Luc DNA immunization on the initiation and growth of the primary focus. Orthotopically and ectopically implanted 4T1luc2 cells exhibit similar growth rate of the primary focus (A); Ectopic implantation of 5000 4T1luc2 cells per site results in the highest growth rate and the highest viability of the cells in the primary focus (B). Site of implantation and the number of implanted cells is depicted on the right. Each point demonstrates the results of two to four independent observations.

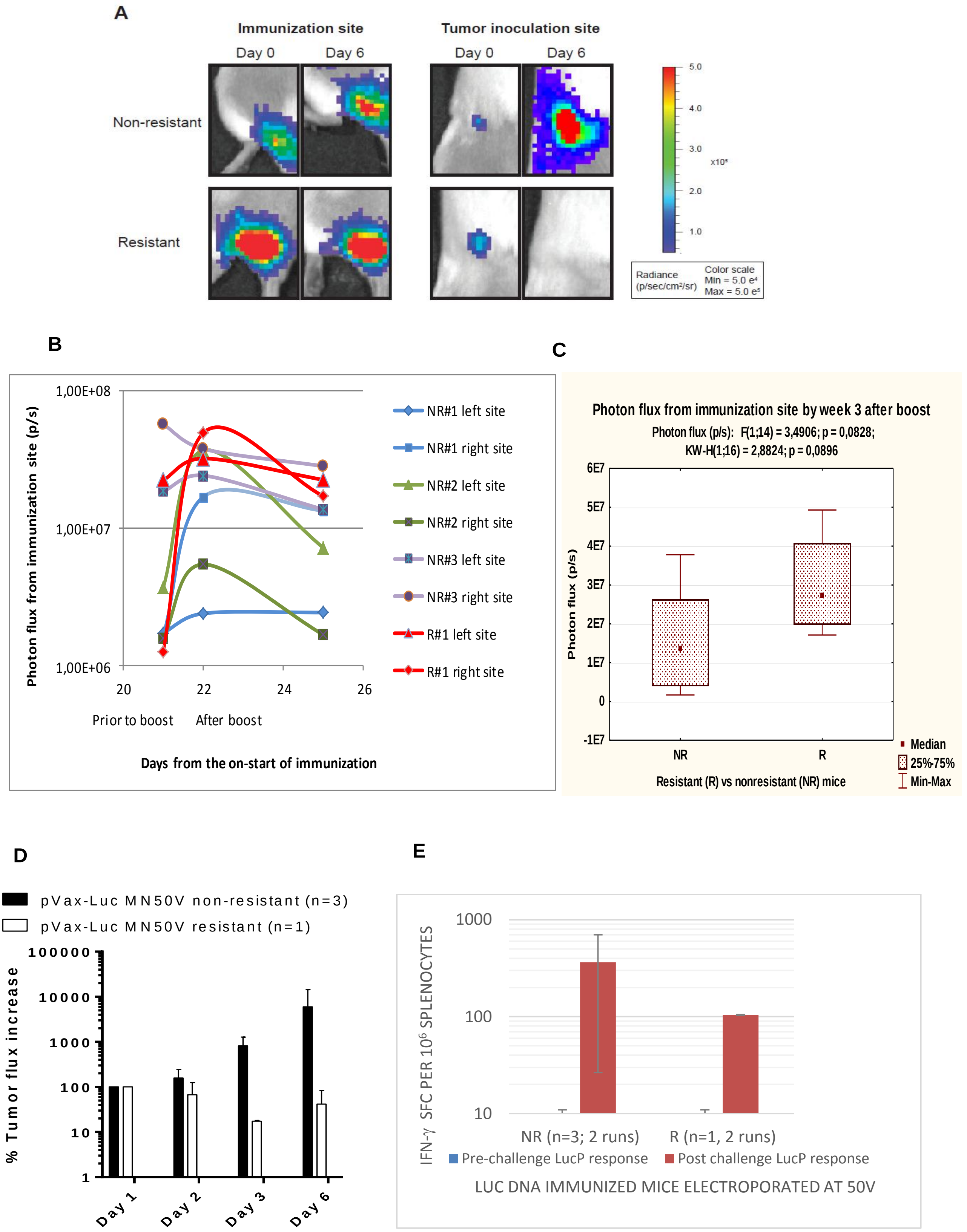

Supplementary Fig. 4 Initiation of 4T1luc2 adenocarcinomas in BALB/c mice DNA-immunized with Luc DNA by intradermal injections followed by low voltage electroporation. BALB/c mice were immunized with Luc DNA followed by electroporation with a BEX device with driving pulses of 50V. Two weeks post boost immunization, mice were challenged with 5 x 10^3 4T1luc2 cells. BLI was performed directly after and on days 1, 2, 3 and 6 post the implantation. Sites of immunization with Luc DNA (left) and of implantation of 4T1luc2 cells (right) in immunized mice resistant and not resistant to tumor initiation at days 0 and 6 post the implantation (A); Total flux from

immunization sites prior to and post Luc DNA boost in mice resistant (R) and nonresistant (NR) to tumor challenge in photons/sec (B) with statistical evaluation (C); Luminescent signal from 4T1luc2 implantation sites visualized as percent change of the total flux compared to the level assessed directly after the implantation ± STDV (D); *In vitro* IFN-γ response of PBMC or splenocytes of Luc DNA mice to stimulation with peptide GFQSMYTFV representing the immunodominant CTL epitope of luciferase (LucP) before and after 4T1luc2 cell challenge± STDV (E).

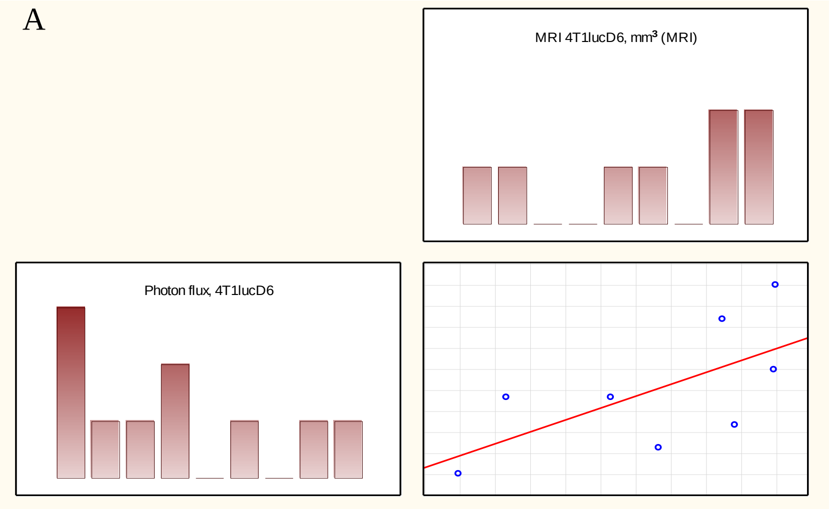

$r^2 = 0{,}4150$; $r = 0{,}6442$; $p = 0{,}0847$

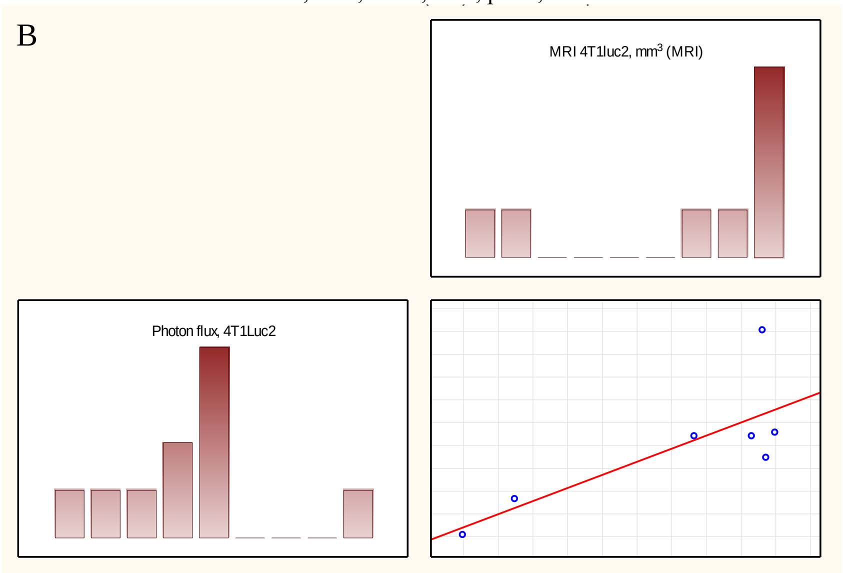

$r^2 = 0{,}5921$; $r = 0{,}7695$; $p = 0{,}0431$

Supplementary Fig. 5 Correlation of tumor size evaluated by MRI (mm^3) and by BLI (photon flux) for tumors generated by 4T1lucD6 (A; R=0,6442, p=0,085) and 4T1Luc2 cell clones (B; R=0,77,p=0,043).
